# Heterogeneous off-target impact of ion-channel deletion on intrinsic properties of hippocampal model neurons that self-regulate calcium

**DOI:** 10.3389/fncel.2023.1241450

**Published:** 2023-10-10

**Authors:** Sunandha Srikanth, Rishikesh Narayanan

**Affiliations:** ^1^Cellular Neurophysiology Laboratory, Molecular Biophysics Unit, Indian Institute of Science, Bangalore, India; ^2^Undergraduate Program, Indian Institute of Science, Bangalore, India

**Keywords:** degeneracy, heterogeneity, homeostasis, ion channels, intrinsic properties, intrinsic plasticity, knockout, theta oscillations

## Abstract

How do neurons that implement cell-autonomous self-regulation of calcium react to knockout of individual ion-channel conductances? To address this question, we used a heterogeneous population of 78 conductance-based models of hippocampal pyramidal neurons that maintained cell-autonomous calcium homeostasis while receiving theta-frequency inputs. At calcium steady-state, we individually deleted each of the 11 active ion-channel conductances from each model. We measured the acute impact of deleting each conductance (one at a time) by comparing intrinsic electrophysiological properties before and immediately after channel deletion. The acute impact of deleting individual conductances on physiological properties (including calcium homeostasis) was heterogeneous, depending on the property, the specific model, and the deleted channel. The underlying many-to-many mapping between ion channels and properties pointed to ion-channel degeneracy. Next, we allowed the other conductances (barring the deleted conductance) to evolve towards achieving calcium homeostasis during theta-frequency activity. When calcium homeostasis was perturbed by ion-channel deletion, post-knockout plasticity in other conductances ensured resilience of calcium homeostasis to ion-channel deletion. These results demonstrate degeneracy in calcium homeostasis, as calcium homeostasis in knockout models was implemented in the absence of a channel that was earlier involved in the homeostatic process. Importantly, in reacquiring homeostasis, ion-channel conductances and physiological properties underwent heterogenous plasticity (dependent on the model, the property, and the deleted channel), even introducing changes in properties that were not directly connected to the deleted channel. Together, post-knockout plasticity geared towards maintaining homeostasis introduced heterogenous off-target effects on several channels and properties, suggesting that extreme caution be exercised in interpreting experimental outcomes involving channel knockouts.

## Introduction

Existing literature points to evidence that deletion of ion-channel conductances could result in compensation of certain functions by significant alteration to the expression and localization of other channels/receptors (Chen et al., 2006, 2010; Andrasfalvy et al., 2008; Nerbonne et al., 2008; Marder, 2011; O’Leary et al., 2014). In a scenario where the intrinsic properties of a neuron undergo plasticity towards maintaining calcium homeostasis with behaviorally relevant afferent activity, how does the neuron respond to perturbations involving removal of a specific channel? Would neurons still be capable of achieving calcium homeostasis when these channels are absent? If yes, does maintenance of calcium homeostasis imply complete compensation of function, in terms of restoring neuronal intrinsic properties and firing patterns? Or would the emergence of calcium homeostasis through updates to ion-channel conductances result in altered intrinsic properties? There have been studies that have addressed related questions using a variety of different experimental and theoretical techniques (Chan et al., 2007; Grashow et al., 2010; Ransdell et al., 2012; Zhao and Golowasch, 2012; O’Leary et al., 2014; Franci et al., 2020; Yang et al., 2022). In this study, we focus on a heterogeneous population of hippocampal model neurons receiving physiologically relevant activity. We systematically evaluate the impact of deleting each of the several ion channels in each model neuron in the heterogeneous population. We focus on addressing the questions from the perspective of impact immediately after channel deletion, of whether calcium homeostasis is achievable after deletion, and if compensation towards achieving calcium homeostasis affect neuronal intrinsic properties across all these models.

**Figure 1:**
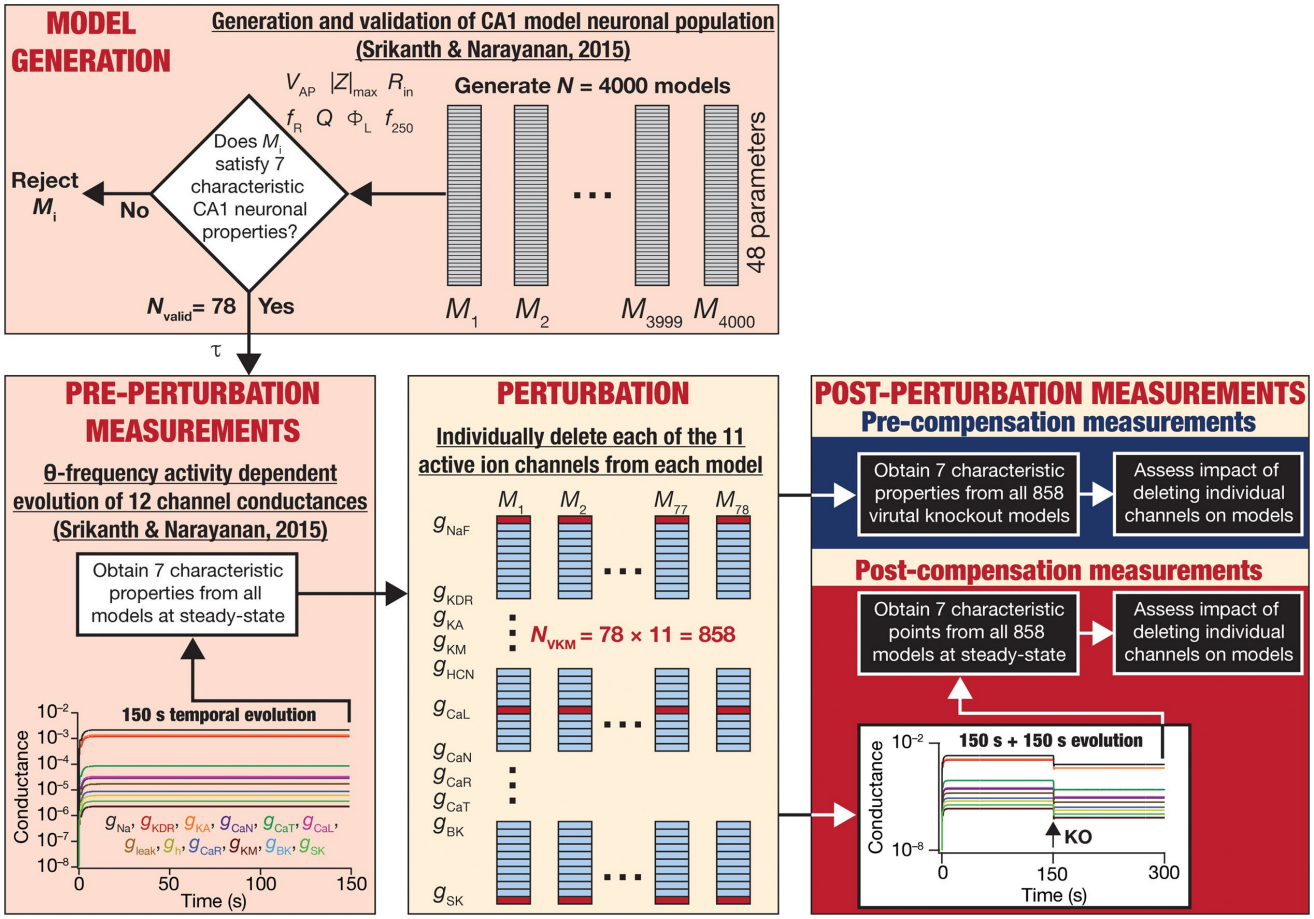
Overall experimental plan of the study. 4,000 models spanning 48 parameters (Table 1) were generated as part of a stochastic search algorithm. Seven characteristic physiological properties — firing rate at 250 pA (*f*_250_; Hz), action potential amplitude (*V*_AP_; mV), input resistance (*R*_in_; MΩ), maximum impedance amplitude (|*Z*|_max_; MΩ), resonance frequency (*f*_R_; Hz), strength of resonance (*Q*) and total inductive phase (Φ_L_; rad.Hz) — were obtained and validated against their electrophysiological counterparts (Table 2). Parameters from the 78 valid models that satisfied all electrophysiological constraints were used for driving calcium homeostasis in 78 distinct models receiving theta frequency afferent activity. At steady-state where calcium homeostasis was achieved (after 150 s of calcium-driven plasticity in intrinsic conductances with theta-frequency afferent activity), the 7 characteristic physiological properties were obtained for each of the 78 neurons (referred to as pre-perturbation measurements). Then, for each neuron, one of the 11 active ion channels was deleted by setting that specific conductance to zero, yielding a total of virtual knockout models (*N*_VKM_ = 858). Two sets of measurements were obtained after the knockout: one (pre-compensation measurements) immediately after the knockout, and another (post-compensation measurements) after reaching steady state (additional 150 s) following temporal evolution of the 10 other conductances towards achieving calcium homeostasis. Theta-frequency afferent activity continued for the post-knockout evolution period as well. The physiological properties recorded at the pre- and post-compensation periods were compared with those obtained before knockout (pre-perturbation) to assess the impact of deleting individual channels and of post-deletion compensation (towards achieving calcium homeostasis).

We allowed calcium levels and conductance values to attain steady state in a heterogeneous population of hippocampal neuronal models (Srikanth and Narayanan, 2015). Importantly, these neurons received physiologically relevant theta frequency afferent activity during the evolution of conductance values. At steady-state of evolution of the conductances and calcium, we “knocked out” a particular conductance (Figure 1). Upon knockout of individual channels, the other conductances were allowed to evolve towards achieving calcium homeostasis while the neuron continued to receive the same theta-frequency afferent activity pattern. We repeated this procedure for each of the 11 active channel conductances in our models and assessed changes in the intrinsic properties of neurons before and after post-knockout plasticity towards achieving calcium homeostasis. This formulation allowed us to assess whether calcium homeostasis was perturbed by channel knockout and if post-knockout calcium homeostasis was achievable through plasticity in other channel conductances.

We computed several physiological properties at three time points: the end of evolution of the full model (pre-perturbation), immediately after knockout of individual channels (pre-compensation), and finally after post-knockout plasticity in the other conductances (post-compensation; Figure 1). Measuring the impact of channel knockouts at three timepoints allowed us to assess the specific nature of compensation effectuated by post-knockout calcium homeostasis in a neuron receiving physiologically relevant activity patterns. Overall, this systematic simulation design involving the tracking of several models to different ion-channel deletions using a variety of measurements allowed us to probe cross-dependencies of outcomes spanning different models, ion channels, and physiological properties.

Our results indicate that the acute impact of knocking out individual conductances on physiological properties was heterogeneous, depending on the measurement, the specific model, and the channel that was deleted. In addition, the perturbation in calcium levels (during theta stimulation) was variable across models in a manner that was also dependent on the channel that was knocked out. When calcium homeostasis was perturbed by ion-channel deletion, post-knockout calcium-dependent plasticity in other conductances restored calcium concentration to the intended target value. These observations indicate the resilience of calcium homeostasis to deletion of individual channels and provide evidence for the expression of degeneracy in achieving calcium homeostasis. Specifically, post-knockout calcium homeostasis in these models was achieved in the absence of one of the channels that was involved in achieving calcium homeostasis before the deletion. These observations point to different components (and their plasticity) being involved in achieving homeostasis in the same neuron. As degeneracy is defined as the ability of disparate structural components to achieve the same function (Edelman and Gally, 2001; Rathour and Narayanan, 2019; Goaillard and Marder, 2021), these results provide a demonstration for the expression of degeneracy in achieving calcium homeostasis through recruitment of different sets of ion-channel conductances (in the same neuron undergoing different channel deletions).

Importantly, we found that post-knockout plasticity was heterogeneous across different models and spanned several channels, together implying heterogenous off-target effects on other channels that were not deleted. Specifically, post-knockout plasticity in other conductances (towards achieving calcium homeostasis) was dependent on the specific model as well as the channel that was deleted in that model. This form of homeostatic intrinsic plasticity was also dependent on the specific intrinsic measurement in question. Consequently, post-knockout plasticity in ion channels and intrinsic properties of the neuron was variable across different models, even for the knockout of the same channel. Furthermore, as different properties exhibited pronounced heterogeneities in knockout-driven plasticity, these observations point to dissociation of functional homeostasis where calcium homeostasis does not imply homeostasis of all functional properties. Our analyses underscore the need for caution in interpreting observations from experiments involving channel knockouts. Such interpretations should be carefully done to account for heterogeneities in channel expression profiles and heterogeneities in post-knockout plasticity towards maintenance of homeostasis.

## Materials and methods

The models used here were generated through an unbiased multi-parametric multi-objective stochastic search (MPMOSS) process described earlier (Srikanth and Narayanan, 2015). The models and physiological properties employed here, the input activity patterns (theta oscillations), and the procedure for implementing calcium homeostasis were identical to the earlier study (Srikanth and Narayanan, 2015). These procedures from (Srikanth and Narayanan, 2015) are described in detail below, followed by the methodology to delete individual channels and assess acute vs. chronic impact of channel deletion (Figure 1).

### Neuronal model and ion channels

Our neuronal model was a single compartmental cylinder with diameter (*d*) of 100 μm and length (*L*) of 100 μm. The specific membrane resistance (*R*_m_), defined as the reciprocal of the leak-channel conductance ( $g_{\mathrm{leak}}$ =1/*R*_m_), was set at 35 kΩ.cm^2^ and specific membrane capacitance (*C*_m_) was 1 μF/cm^2^, together setting the passive input resistance (*R*_in_) at ~111 MΩ (*R*_m_/π*dL*) and the passive membrane time constant at 35 ms (*R*_m_*C*_m_; Narayanan and Johnston, 2007, 2008). We incorporated 11 active ion channels into our model (total 12 ion channels including the leak channels): fast sodium (NaF), delayed-rectifier potassium (KDR), *A*-type potassium (KA), *M*-type potassium (KM), *T*-type calcium (CaT), *R*-type calcium (CaR), *N*-type calcium (CaN), *L*-type calcium (CaL), hyperpolarization-activated cyclic nucleotide gated channel (HCN or *h*), small conductance (SK) and big conductance calcium-activated potassium (BK) channels. The channel kinetics for NaF, KDR and KA were obtained from (Hoffman et al., 1997; Migliore et al., 1999), for CaT from (Shah et al., 2011), for KM from (Migliore et al., 2006), for CaR and CaL from (Magee and Johnston, 1995; Poirazi et al., 2003), CaN and SK from (Migliore et al., 1995), for HCN from (Magee, 1998; Poolos et al., 2002), and for BK from (Moczydlowski and Latorre, 1983). The equilibrium potentials for K^+^ and Na^+^ ions were set at −90 mV and + 55 mV, respectively. The reversal potential for the HCN channel was −30 mV. Current through all calcium channels (CaT, CaR, CaN, and CaL) was modeled using the Goldman-Hodgkin-Katz (GHK) formulation (Goldman, 1943; Hodgkin and Katz, 1949) to account for the strong nonlinearities associated with the steep concentration gradient in the calcium ion. In updating the calcium currents using the GHK current equation, cytosolic calcium ion concentration was continually updated to account for the activity of all calcium on and off mechanisms (see below).

### Synaptic receptors

A canonical synapse consisting of colocalized *N*-methyl D-aspartate receptor (NMDAR) and 2-amino-3-(5-methyl-3-oxo-1,2- oxazol-4-yl) propanoic acid receptor (AMPAR) was introduced to this single-compartmental model (Narayanan and Johnston, 2010). The NMDAR current was modeled as a combination of three ionic currents (Ca^2+^, Na^+^ and K^+^) using the GHK formulation:


(1) $$I_{\mathrm{NMDAR}}\left(v,t\right)=I_{\mathrm{NMDAR}}^{Na}\left(v,t\right)+I_{\mathrm{NMDAR}}^{K}\left(v,t\right)+I_{\mathrm{NMDAR}}^{Ca}\left(v,t\right)$$

where,


(2) $$\begin{array}{l} I_{\mathrm{NMDAR}}^{Na}\left(v,t\right)=\\ P_{\mathrm{NMDAR}}P_{Na}MgB\left(v\right)\frac{vF^{2}}{RT}\left(\frac{{\left[Na\right]}_{i}-{\left[Na\right]}_{o}\exp\left(-\frac{vF}{RT}\right)}{1-\exp\left(-\frac{vF}{RT}\right)}\right) \end{array}$$

(3) $$\begin{array}{l} I_{\mathrm{NMDAR}}^{K}\left(v,t\right)=\\ P_{\mathrm{NMDAR}}P_{K}MgB\left(v\right)\frac{vF^{2}}{RT}\left(\frac{{\left[K\right]}_{i}-{\left[K\right]}_{o}\exp\left(-\frac{vF}{RT}\right)}{1-\exp\left(-\frac{vF}{RT}\right)}\right) \end{array}$$

(4) $$\begin{array}{l} I_{\mathrm{NMDAR}}^{Ca}\left(v,t\right)=\\ P_{\mathrm{NMDAR}}P_{Ca}MgB\left(v\right)\frac{4vF^{2}}{RT}\left(\frac{{\left[Ca\right]}_{i}-{\left[Ca\right]}_{o}\mathrm{ex}p\left(-\frac{2vF}{RT}\right)}{1-\exp\left(-\frac{2vF}{RT}\right)}\right) \end{array}$$

where *P*_NMDAR_ represented the maximum permeability of the NMDAR; *P_Ca_* = 10.6, *P_Na_* = 1, *P_K_* = 1 (Mayer and Westbrook, 1987; Canavier, 1999). Extracellular and intracellular concentrations of ions were (in mM): [*Na*]*_i_* = 18, [*Na*]*_o_* = 140, [*K*]*_i_* = 140, [*K*]*_o_* = 5, [*Ca*]*_i_* = 50 × 10^−6^ (resting concentration) [*Ca*]*_o_* = 2. [*Ca*]*_i_* was continually updated to account for calcium influx into the cytosol through the different receptors and channels as well as changes in cytosolic calcium due to calcium buffers, pumps, and diffusion (see below). The ionic concentrations of the Na^+^ and K^+^ ions ensured that their equilibrium potentials were the same as those set for the voltage-gated ion channels: +55 mV for Na^+^ and − 90 mV for K^+^ ions. *MgB(v)* was a voltage-dependent function that accounted for the magnesium-dependent pore blockade of NMDAR receptors (Jahr and Stevens, 1990):


(5) $$MgB\left(v\right)={\left(1+\frac{{\left[\mathrm{Mg}\right]}_{o}\exp\left(-0.062v\right)}{3.57}\right)}^{-1}$$

where [*Mg*]*_o_* = 2 mM. The GHK formulation was used to model NMDARs so that the calcium current $I_{\mathrm{NMDAR}}^{Ca}\left(v,t\right)$ through the NMDARs can be separated towards contributing to changes in cytosolic calcium concentration.

We used the GHK formulation for AMPARs to be consistent with the NMDAR formulation and provide a more accurate *I–V* relationship compared to a Ohmic formulation based on the receptor reversal potential. Specifically, the total AMPAR current was the sum of currents carried by Na^+^ and K^+^ ions:


(6) $$I_{\mathrm{AMPAR}}\left(v,t\right)=I_{\mathrm{AMPAR}}^{Na}\left(v,t\right)+I_{\mathrm{AMPAR}}^{K}\left(v,t\right)$$

where,


(7) $$I_{\mathrm{AMPAR}}^{Na}\left(v,t\right)=P_{\mathrm{AMPAR}}P_{Na}\frac{vF^{2}}{RT}\left(\frac{{\left[Na\right]}_{i}-{\left[Na\right]}_{o}\exp\left(-\frac{vF}{RT}\right)}{1-\exp\left(-\frac{vF}{RT}\right)}\right)$$

(8) $$I_{\mathrm{AMPAR}}^{K}\left(v,t\right)=P_{\mathrm{AMPAR}}P_{K}\frac{vF^{2}}{RT}\left(\frac{{\left[K\right]}_{i}-{\left[K\right]}_{o}\exp\left(-\frac{vF}{RT}\right)}{1-\exp\left(-\frac{vF}{RT}\right)}\right)$$

where *P*_AMPAR_ represented maximum permeability, with the relative sodium and potassium permeabilities equal for the AMPAR (*P_Na_* = *P_K_*; Dingledine et al., 1999). NMDAR:AMPAR ratio (*NAR*) was used to define the relationship between AMPAR and NMDAR permeabilities:


(9) $$P_{\mathrm{NMDAR}}=NAR\times P_{\mathrm{AMPAR}}$$

The default value of *NAR* was 1.5.

### Calcium dynamics

As the model involves regulation of calcium concentration, we incorporated an array of calcium handling mechanisms into the model. These mechanisms accounted for pumps on the plasma membrane and the endoplasmic reticular membrane, plasma membrane calcium channels (PMCC) which include the voltage-gated calcium channels (VGCC: *L*-, *T*-, *R*- and *N*-type calcium channels) and NMDAR, radial diffusion of calcium (across 4 concentric annuli) in our neuronal model, and calcium buffers (Ashhad and Narayanan, 2013; Srikanth and Narayanan, 2015). Together, the evolution of cytosolic calcium was modeled as (Sneyd et al., 1995; Fink et al., 2000):


(10) $$\begin{array}{l} \frac{\partial {\left[Ca\right]}_{i}}{\partial t}=\\ D_{Ca}\nabla^{2}{\left[Ca\right]}_{i}+\beta \left(J_{\mathrm{leak}}-J_{\mathrm{SERCA}}\right)+R_{buf}+J_{\mathrm{PMCC}}-J_{\mathrm{pump}} \end{array}$$

where *D*_Ca_ represented the calcium diffusion constant whose value was taken from (Allbritton et al., 1992; Klingauf and Neher, 1997); β governed the density of leak channels and SERCA pumps on the endoplasmic reticulum (ER) membrane; *J*_PMCC_, *J*_SERCA_, *R*_buf_, *J*_pump_ and *J*_leak_ were calcium flux due to PMCC, sarcoendoplasmic reticulum calcium ATPase (SERCA) pumps, static calcium buffers, plasma membrane pumps, and ER calcium leak channels, respectively. As calcium diffused radially across 4 concentric annuli, cytosolic calcium concentration for regulatory purposes was that of the outermost annulus (Carnevale and Hines, 2006; Ashhad and Narayanan, 2013).

Turning to the individual components in Eq. (10), cytosolic calcium influx through the ER leak channels was given by (Fink et al., 2000; Ashhad and Narayanan, 2013):


(11) $$J_{\mathrm{leak}}=L\left(1-\frac{{\left[Ca\right]}_{i}}{{\left[Ca\right]}_{ER}}\right)\;mM/\mathrm{ms}$$

where the leak constant *L* was chosen such that the net calcium flux through ER leak channels was zero at resting state (−65 mV). [*Ca*]_ER_, the ER calcium concentration was set at 400 μM. The contribution of PMCCs to cytosolic calcium concentration was given by (Poirazi et al., 2003; Ashhad and Narayanan, 2013):


(12) $$J_{\mathrm{PMCC}}=-\frac{I_{Ca}\times \pi \times \mathrm{diam}}{2F}\;mM/\mathrm{ms}$$

where *I*_Ca_ represented calcium current through both plasma membrane calcium channels (VGCCs and NMDARs; Eq. 4), *diam* is the diameter of the compartment, *F* is the Faraday constant. The negative sign accounts for the inward nature of *I*_Ca_, thereby providing an influx of calcium into the cytosol with increase in the magnitude of *I*_Ca_. The pumping activity of the SERCA pump was represented by the flux (Fink et al., 2000; Ashhad and Narayanan, 2013):


(13) $$J_{\mathrm{SERCA}}=V_{\text{max}}\frac{{{\left[Ca\right]}_{i}}^{2}}{{{\left[Ca\right]}_{i}}^{2}+{K_{p}}^{2}}\;mM/\mathrm{ms}$$

where *V*_max_ represented the average amplitude of pump uptake (1 × 10^−4^ mM/ms) and *K*_p_ was the dissociation constant associated with the binding of calcium to the SERCA pump (0.27 μM).

*J*_pump_, representing calcium extrusion through plasma membrane calcium pumps, was modeled as a flux that was dependent on a threshold on the value of cytosolic calcium. Explicitly, the pumps were inactive below a threshold value of [*Ca*]_i_ ([*Ca*]_crt_), above which the flux was linearly dependent on [*Ca*]_i_ (Fink et al., 2000):


(14) $$J_{\mathrm{pump}}=\{\;\begin{array}{ccc} \gamma \left({\left[Ca\right]}_{i}-{\left[Ca\right]}_{crt}\right) & : & {\left[Ca\right]}_{i}\geq {\left[Ca\right]}_{crt}\\ \; & \; & \;\\ 0 & : & \mathrm{otherwise} \end{array}$$

where [*Ca*]*_crt_* (0.2 μM) and γ (8 μm/s) were parameters that governed pump extrusion (Herrington et al., 1996; Fink et al., 2000; Ashhad and Narayanan, 2013). Stationary buffers were incorporated into the model as follows (Ashhad and Narayanan, 2013):


(15) $$R_{buf}=-k_{\mathrm{on}}{\left[Ca\right]}_{i}\left[B_{buf}\right]+k_{\mathrm{off}}\left[Ca\;B_{buf}\right]$$

(16) $$\frac{d\left[B_{buf}\right]}{dt}=\frac{d\left[Ca\;B_{buf}\right]}{dt}=R_{buf}$$

(17) $$K_{buf}=\frac{k_{\mathrm{off}}}{k_{\mathrm{on}}}$$

where [*B*_buf_] (=450 μM) and [*Ca B*_buf_] were free-buffer and calcium-bound-buffer concentrations. The on and off rate constants for calcium binding to the buffer were represented by *k*_on_ and *k*_off_, respectively. Eq. (16) is a pseudo state approximation, considering free- and calcium-bound buffers to be in equilibrium. *K*_buf_ = 10 μM (Klingauf and Neher, 1997; Fink et al., 2000; Ashhad and Narayanan, 2013).

### Physiological properties

A set of standard intrinsic physiological properties were used to characterize and validate neuronal models (Narayanan and Johnston, 2008; Srikanth and Narayanan, 2015). The sub- and supra-threshold measures for neuronal excitability were firing rate of the model at 250 pA (*f*_250_; Hz), amplitude of the action potential (*V*_AP_; mV), input resistance (*R*_in_; MΩ), and maximum impedance amplitude computed from the chirp stimulus (|*Z*|_max_; MΩ). Neuronal intrinsic response dynamics were assessed by computing resonance frequency (*f*_R_; Hz), resonance strength (*Q*) and total inductive phase (Φ_L_; rad.Hz).

Firing rate *f*_250_ was computed as the number of action potentials elicited by a model to a 250 pA current (for 500 ms; count was multiplied by 2 to obtain firing rate in Hz). Action potential amplitude was the difference between resting membrane potential (RMP = −65 mV) and the peak action-potential voltage. Currents (*I*) of −50 pA to 50 pA, in steps of 10 pA (11 values), were injected for 500 ms into the cell. Steady-state voltage deflection (*V*) from RMP was measured for each current value. Input resistance was measured as the slope of the linear fit to the *V–I* plot that was constructed by these 11 points.

We measured the voltage response $V\left(t\right)$ of the model to a chirp current $I\left(t\right)$ , a sinusoid of constant amplitude of 50 pA, with frequency increasing linearly from 0 to 25 Hz over a period of 25 s. Impedance [ $Z\left(f\right)$ ] was computed as the ratio of the Fourier transform of the voltage response [ $V\left(f\right)$ ] by the Fourier transform of the chirp current [ $I\left(f\right)$ ]. Impedance amplitude profile as a function of frequency was calculated as the magnitude of the complex quantity $Z\left(f\right)$ :


(18) $$\left|Z\left(f\right)\right|=\sqrt{{\left(Re\left(Z\left(f\right)\right)\right)}^{2}+{\left(Im\left(Z\left(f\right)\right)\right)}^{2}}$$

where Re ( $Z\left(f\right)$ ) and Im ( $Z\left(f\right)$ ) represented the real and imaginary components of $Z\left(f\right)$ . The frequency at which | $Z\left(f\right)$ | reached its maximum value was defined as the resonance frequency, *f*_R_. The maximal value of impedance amplitude, which would be achieved at *f*_R_ (i.e., | $Z\left(f_{R}\right)$ |), was defined as maximum impedance amplitude, |*Z*|_max_. Resonance strength (*Q*) was computed as the ratio of |*Z*|_max_ and | $Z\left(0.5\;\mathrm{Hz}\right)$ |. Impedance phase profile ( $\phi \left(f\right)$ ) was computed the phase associated with $Z\left(f\right)$ :


(19) $$\phi \left(f\right)=\tan^{-1}\frac{Im\left(Im\;Z\left(f\right)\right)}{Re\left(Z\left(f\right)\right)}$$

Total inductive phase $\Phi_{L}$ was the area under the inductive (i.e., positive) part of phase curve *ϕ*(*f*) (Narayanan and Johnston, 2008):


(20) $$\Phi_{L}=\underset{\phi \left(f\right)>0}{\int }\phi \left(f\right)\;df$$

### Multi-parametric multi-objective stochastic search

A multi-parametric multi-objective stochastic search (MPMOSS) procedure (Bhalla and Bower, 1993; Foster et al., 1993; Goldman et al., 2001; Golowasch et al., 2002; Prinz et al., 2003, 2004; Achard and De Schutter, 2006; Tobin et al., 2006; Reid et al., 2007; Hobbs and Hooper, 2008; Weaver and Wearne, 2008; Taylor et al., 2009; Rathour and Narayanan, 2012b, 2014; Srikanth and Narayanan, 2015) was used to sample and validate neuronal models that satisfied characteristic physiological properties of CA1 pyramidal neurons. A total of 4,000 unique models were randomly generated by sampling independent uniform distributions associated with each of the 48 model parameters (Table 1). We validated these models by requiring all 7 of their physiological properties to have their values within respective electrophysiological bounds for CA1 pyramidal neurons (Table 2). We found 78 out of the 4,000 (~2%) randomly generated models to be valid, whereby they satisfied all 7 electrophysiological bounds (Table 2). The 78 models were designated as valid models, and all analyses were performed on these valid models (Figure 1).

**Table 1:** Parameters, their default values in the base model, and the range over which random sampling was performed during the MPMOSS procedure.

	Parameter, unit	Symbol	Default value	Testing range
**Passive parameters**				
1	Specific membrane resistance, kΩ.cm^2^	*R*_m_	35	30–40
2	Specific membrane conductance, μF/cm^2^	*C*_m_	1	0.5–1.5
**Na channel parameters**				
3	Maximal conductance, S/cm^2^	*Na-g*	0.007	0.005–0.01
4	Inactivation time constant, ms	*Na-τ*_h_	2.34	1.87–2.81
5	Activation time constant, ms	*Na-τ*_m_	0.163	0.13–0.20
6	Slow inactivation time constant, ms	*Na-τ*_s_	106.1	84.88–127.32
7	*V*_1/2_ inactivation, mV	*Na-V*_h_	−45	−47 to −43
8	*V*_1/2_ activation, mV	*Na-V*_m_	−30	−32 to −28
9	*V*_1/2_ slow inactivation, mV	*Na-V_s_*	−60	−62 to −58
**KDR channel parameters**				
10	Maximal conductance, S/cm^2^	*DR-g*	0.003	0.001–0.005
11	Activation time constant, ms	*DR-τ*_n_	222.9	111.45–445.8
12	*V*_1/2_ activation, mV	*DR-V*_n_	13	10–15
**KA channel parameters**				
13	Maximal conductance, S/cm^2^	*A-g*	0.008	0.001–0.01
14	Inactivation time constant, ms	*A-τ*_l_	2	1–4
15	Activation time constant, ms	*A-τ*_n_	0.137	0.086–0.43
16	*V*_1/2_ inactivation, mV	*A-V*_l_	−56	−60 to −50
17	*V*_1/2_ activation, mV	*A-V*_n_	11	8–15
**CaT channel parameters**				
18	Maximal conductance, mS/cm^2^	*T-g*	0. 1	0.05–0. 2
19	Inactivation time constant, ms	*T-τ*_h_	31.02	10.24–46.53
20	Activation time constant, ms	*T-τ*_m_	0.858	0.43–1.72
21	*V*_1/2_ inactivation, mV	*T-V*_h_	−75	−80 to −70
22	*V*_1/2_ activation, mV	*T-V*_m_	−28	−25 to −15
**HCN channel parameters**				
23	Maximal conductance, mS/cm^2^	*h-g*	0.08	0.005–0.05
24	Activation time constant, ms	*h-τ*_l_	28.5	20.52–71.25
25	*V*_1/2_ activation, mV	*h-V*_l_	−81	−85 to −70
**CaL channel parameters**				
26	Maximal conductance, μS/cm^2^	*L-g*	100	50–200
27	Activation time constant, ms	*L-τ*_m_	0.189	1.8–7.2
28	*V*_1/2_ activation, mV	*L-V*_a_	−27.01	−30 to −24
**CaR channel parameters**				
29	Maximal conductance, μS/cm^2^	*R-g*	100	50–200
30	Inactivation time constant, ms	*R-τ*_h_	12.7	6.35–25.4
31	Activation time constant, ms	*R-τ*_m_	0.221	0.11–0.442
32	*V*_1/2_ inactivation, mV	*R-V*_h_	−39	−43 to −35
33	*V*_1/2_ activation, mV	*R-V*_m_	3	−2–7
**SK channel parameters**				
34	*Ca*_1/2_ activation, nM	*SK-Ca*	140	110–180
35	Maximal conductance, μS/cm^2^	*SK-g*	1	0.5–5
36	Activation time constant, ms	*SK-τ*	196.8	98.4–393.6
**BK channel parameters**				
37	Maximal conductance, μS/cm^2^	*BK-g*	1	0.5–5
38	Slope of Ca activation (mM)	*BK-k*_1_	4.8 × 10^−4^	2.8 × 10^−4^–6.8 × 10^−4^
39	*Ca*_1/2_ activation (nM)	*BK-k*_2_	0.13	0.08–0.18
40	Activation time constant, ms	*BK-τ*	8.04	4.04–16.08
**KM channel parameters**				
41	Maximal conductance, μS/cm^2^	*M-g*	1	0.5–5
42	Activation time constant, ms	*M-τ*	6,662	3,331–13,323
43	*V*_1/2_ activation, mV	*M-V*	−40	−45 to −35
**CaN channel parameters**				
44	Maximal conductance, μS/cm^2^	*N-g*	100	50–200
45	Inactivation time constant, ms	*N-τ*_h_	1,555	777.5–3,110
46	Activation time constant, ms	*N-τ*_m_	0.942	0.471–1.884
47	*V*_1/2_ inactivation, mV	*N-V*_h_	39	35–44
48	*V*_1/2_ activation, mV	*N-V*_m_	19.88	15–24

The default base values in the table were obtained by hand-tuning a base model (Srikanth and Narayanan, 2015).

**Table 2:** Constraints on physiological properties for declaring a model to be valid using the MPMOSS procedure (Srikanth and Narayanan, 2015).

Physiological property, unit	Lower bound	Upper bound
*f*_250_, Hz	10	35
*V*_AP_, mV	90	110
*R*_in_, MΩ	50	90
|*Z*|_max_, MΩ	50	110
*f*_R_, Hz	2	5.5
*Q*	1.01	1.5
Φ_L_, rad.Hz	0	0.15

These bounds were extracted from experimental recordings (somatic recordings) presented in (Narayanan and Johnston, 2007, 2008; Narayanan et al., 2010).

### Calcium homeostasis

Activity-dependent self-regulation of calcium in our CA1 pyramidal neuron models (Srikanth and Narayanan, 2015) was adapted from a previous model (O’Leary et al., 2014). In this framework, a negative feedback loop involving error in average cytosolic calcium with reference to a target calcium value drives homeostasis by altering a transcription factor, which in turn regulates channel expression profiles. A single transcription factor regulated calcium-dependent transcription of all 12 ion channels. The evolution of the messenger RNA (mRNA) associated with each channel (*m_j_*) was given as:


(21) $$\tau_{j}{\dot{m}}_{j}={\left[Ca\right]}_{tgt}-{\left[Ca\right]}_{i}$$

where $\tau_{j}$ represented the time constant associated with each mRNA evolution, [*Ca*]*_tgt_* (= 200 nM) was the target value of [*Ca*]*_i_*. The right-hand side of Eq. (21) is therefore an error term between the current value of calcium concentration and the target value. Calcium homeostasis was considered to be maintained when this error term was equal to zero (target calcium concentration achieved). The following equation governed the evolution of ion-channel conductances ( $g_{j}$ ), given their respective mRNA (*m_j_*) values:


(22) $$\tau_{g}{\dot{g}}_{j}=m_{j}-g_{j}$$

where $\tau_{g}$ represented the time constant of this evolution, and was set to be identically equal to 10 ms for all 12 conductances ( $g_{Na}$ , $g_{KDR}$ , $g_{KA}$ , $g_{\mathrm{CaN}}$ , $g_{\mathrm{CaT}}$ , $g_{CaL}$ , $g_{\mathrm{leak}}$ , $g_{h}$ , $g_{\mathrm{CaR}}$ , $g_{KM}$ , $g_{BK}$ , $g_{SK}$ ). In an additional set of simulations, we observed that changes to $\tau_{g}$ merely altered the kinetics associated with the evolution, but not the steady-state $g_{j}$ values when homeostasis was achieved. We randomly initialized all *m*_j_ and *g*_j_ to very small values. $\tau_{j}$ values determining the evolution of channel mRNAs were selected from conductance ratios of specific valid models obtained from the MPMOSS procedure. Specifically, $\tau_{j}$ were set using the following ratio relationship (O’Leary et al., 2014):


(23) $$\frac{\tau_{j}}{\tau_{i}}=\frac{g_{i}^{k}}{g_{j}^{k}}$$

where *i* and *j* represented the 12 ion channels, and *k* varied from 1 to 78 (number of valid models).

The $\tau_{i}$ value associated with the sodium conductance was set at 10 ms and all the other $\tau_{i}$ values were computed using specific conductance ratios [from Eq. (23)]. When we changed the specific value of the $\tau_{i}$ (from 10 ms to another value) for the sodium channel and recomputed the other $\tau_{i}$ values, we found the time-course towards reaching steady-state to be altered, but the steady-state values of the conductances remained the same. The dynamics associated with $m_{j}$ and $g_{j}$ were independently assessed for each of the 78 valid models by computing the $\tau_{i}$ values to be dependent on the conductance ratios in these different models (O’Leary et al., 2014). As our parametric search spans parameters beyond conductances (Table 1; the half-maximal activation voltages, time constants of ion channels and passive properties), we ensured that we set our model to reflect these values from the same valid model that was used for computing the $\tau_{i}$ values. Thus, while the conductances from each valid model were used to compute the time constants (Eq. 23), all other parameters from that specific valid model were used to define the channels and other properties of the model that evolved towards calcium homeostasis.

### Evolution of calcium homeostasis with theta oscillatory afferent inputs

Changes in afferent inputs to the model neuron were presented as changes to the activation of synaptic receptors, specifically reflecting directly as changes to AMPAR and NMDAR permeabilities (Eqs. 2–9). We evaluated the evolution of model calcium within the synaptic activation driven by theta (4–10 Hz range) oscillations, which is an activity pattern that is physiologically relevant to hippocampal pyramidal neurons as they receive theta-modulated inputs during exploratory/REM-sleep state (Buzsaki, 2002; Buzsaki and Moser, 2013; Colgin, 2013). Theta oscillations were modeled using a concurrent 8-Hz sinusoidal modulation (Buzsaki et al., 1983; Harvey et al., 2009) of the permeabilities of both AMPAR and NMDAR, with the amplitude of the sinusoidal permeability modulation set as the minimum amplitude required to elicit action potentials in the neuron.

### Virtual knockout analysis: acute versus chronic impact on model physiology

For each of the 78 valid neurons, the model of activity-dependent calcium homeostasis (Eq. 21–23) was employed to evolve all 12 conductances, with 8-Hz theta-frequency oscillations presented as afferent inputs for a 150 s period (Figure 1). The 7 intrinsic properties (*f*_250_, *V*_AP_, *R*_in_, |*Z*|_max_, *f*_R_, *Q* and Φ_L_) were measured at steady-state (at the end of the 150 s period) and were referred to as “Pre-perturbation measurements” (Figure 1). At steady state, one of the 11 active conductances (i.e., except for $g_{\mathrm{leak}}$ ) was “virtually knocked out” (referred to as the perturbation) by making its value to be zero (O’Leary et al., 2014; Rathour and Narayanan, 2014). The 7 intrinsic properties (*f*_250_, *V*_AP_, *R*_in_, |*Z*|_max_, *f*_R_, *Q* and Φ_L_) were recorded a second time immediately after knocking out and were referred to as “Pre-compensation measurements” (Figure 1). As the knockout of the conductance might have altered calcium concentrations in this model, calcium-dependent evolution of the other 11 conductances were resumed towards achieving the unaltered target level of calcium. The post-knockout temporal evolution also was set to proceed for a period of 150 s. The 7 intrinsic properties were recorded for the third time, at the steady state of this post-knockout evolution of calcium and ionic conductances, and were referred to as “Post-compensation measurements” (Figure 1). This procedure was repeated for each of the 11 active conductances (NaF, KDR, KA, KM, HCN, CaL, CaN, CaR, CaT, BK, SK), across each of the 78 models in the neuronal population (Figure 1).

### Simulation details

All simulations were performed in the NEURON simulation environment (Carnevale and Hines, 2006). The resting membrane potential was set at −65 mV and the temperature was 35°C. The integration time constant for all simulations was set at 25 μs. We accounted for temperature dependence of channel kinetics using experimentally measured *Q*_10_ values for the channels in the model. Custom-written code with IGOR Pro (Wavemetrics) was used for performing data analyses and data representation.

## Results

How does deletion of individual ion-channels affect calcium homeostasis and physiological properties of neurons that are endowed with cell-autonomous self-regulation of calcium homeostasis? In addressing this question, we first recognized that the use of a single hand-tuned model would introduce biases in terms of the specific values associated with the ion-channel conductances and other properties (Table 1). Therefore, we used a heterogeneous population of 78 CA1 pyramidal neuron models that were validated against 7 physiological properties (Table 2). We employed the conductance values and other properties from these models to drive temporal evolution dynamics of calcium homeostasis while the neurons received physiologically relevant theta-patterned afferent activity. Calcium was self-regulated by the error in average cytosolic calcium value attained in comparison to a target value of calcium. This error regulated a single transcription factor, which in turn altered the mRNA concentration and channel density of the associated ion-channel. The updated channel densities and associated changes in voltage responses then altered the calcium levels towards achieving calcium homeostasis (O’Leary et al., 2014; Srikanth and Narayanan, 2015).

The impact of blocking or knocking out individual ion channels on different electrophysiological properties has been known in populations of CA1 pyramidal neurons (Nolan et al., 2004; Chen et al., 2006; Narayanan and Johnston, 2008; Rathour and Narayanan, 2012a, 2014, 2019; Anirudhan and Narayanan, 2015; Rathour et al., 2016; Mukunda and Narayanan, 2017; Basak and Narayanan, 2018b, 2020; Seenivasan and Narayanan, 2020; Roy and Narayanan, 2021) as well as in other neuronal subtypes (Mittal and Narayanan, 2018, 2022; Goaillard and Marder, 2021; Mishra and Narayanan, 2021a,b; Roy and Narayanan, 2022), as established from electrophysiological and computational studies. However, the impact of channel deletion on different intrinsic properties in a self-regulating neuronal population geared towards achieving calcium homeostasis has not been assessed. To do this, we devised a strategy (Figure 1) whereby we allowed steady-state attainment of calcium homeostasis (by calcium-driven intrinsic plasticity of all 12 ion channels) in each of the 78 model neurons while they received theta-frequency afferent activity. We measured the seven characteristic intrinsic properties at the end of this temporal evolution (pre-perturbation measurements). We then knocked out a single ion channel (one of the 11 active channels) by setting their conductance value to 0. We computed the 7 physiological properties immediately after channel deletion (pre-compensation measurements). Finally, we allowed continuation of temporal evolution towards achieving calcium homeostasis through changes in the 11 other channels (other than the one that was knocked out) when the neuron received theta-frequency activity. We obtained a final set of 7 the physiological properties (post-compensation measurements) that would provide insights about post-knockout plasticity in these intrinsic properties incurred towards achieving calcium homeostasis (Figure 1).

**Figure 2:**
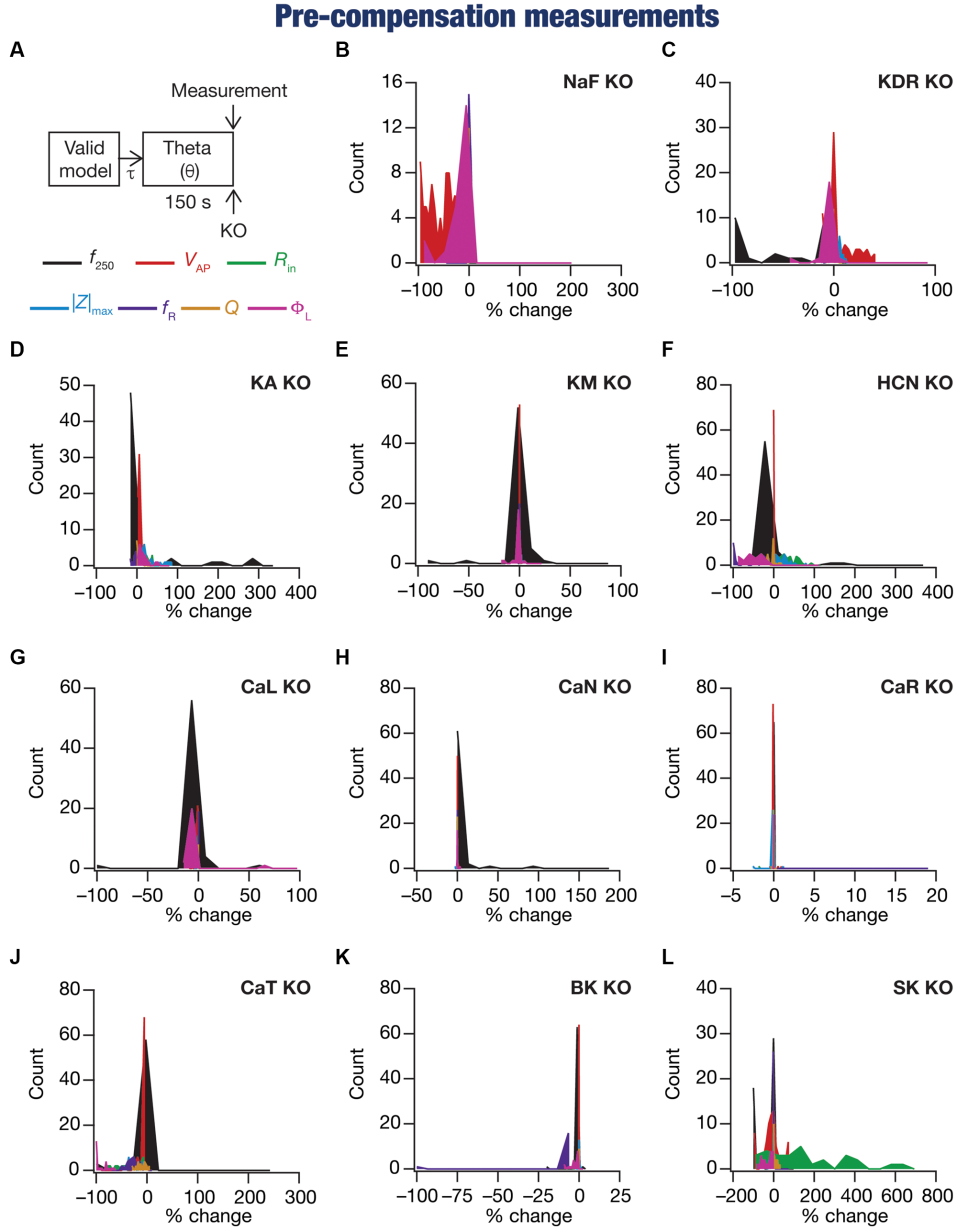
Virtual knockout of the ion channels has differential and variable impact on the seven physiological properties across 78 valid models. **(A)** Experimental design for assessing the effect of virtually knocking out ionic conductances on the seven intrinsic response properties (*f*_250_, *V*_AP_, *R*_in_, |Z|_max_, *f*_R_, *Q*, Φ_L_; color-coded) across 78 neuronal models. **(B–L)** Histogram of percentage changes (pre-compensation measurements compared with pre-perturbation measurements) in the 7 intrinsic response properties in response to knocking out each of the 11 active ionic conductances individually [NaF **(B)**; KDR **(C)**; KA **(D)**; KM **(E)**; HCN **(F)**; CaL **(G)**; CaN **(H)**; CaR **(I)**; CaT **(J)**; BK **(K)**; SK **(L)**].

**Figure 3:**
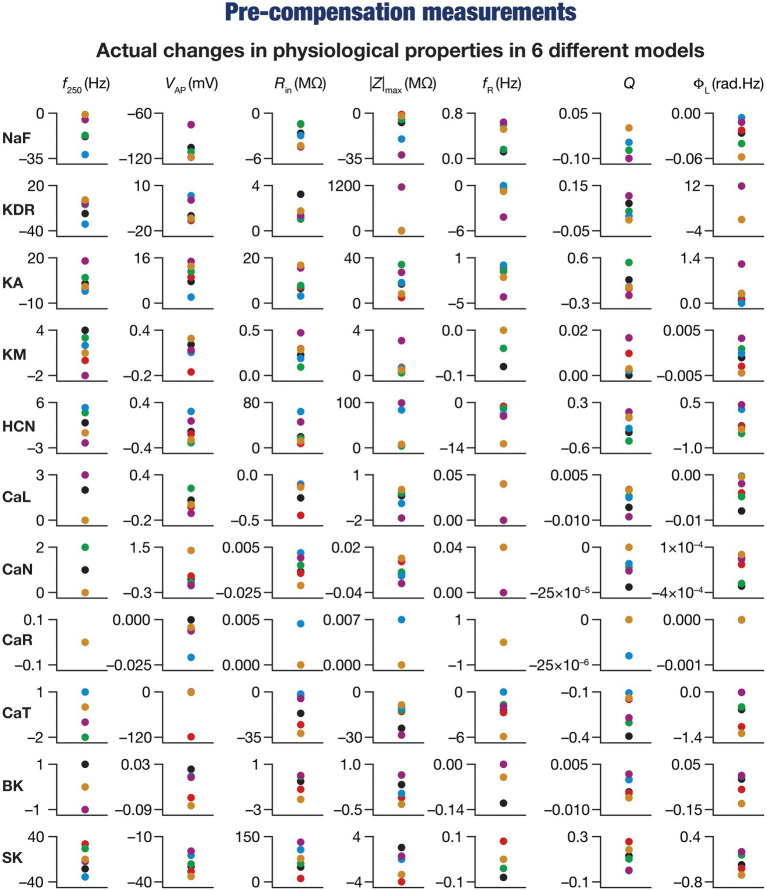
Virtual knockout of the ion channels has differential and variable impact on the seven physiological properties in 6 model neurons. Actual differences in seven intrinsic properties (*f*_250_, *V*_AP_, *R*_in_, |*Z*|_max_, *f*_R_, *Q*, Φ_L_) after virtual knockout of different conductances compared with steady state of theta-dependent evolution (θ) of 6 different valid models (color-coded).

**Figure 4:**
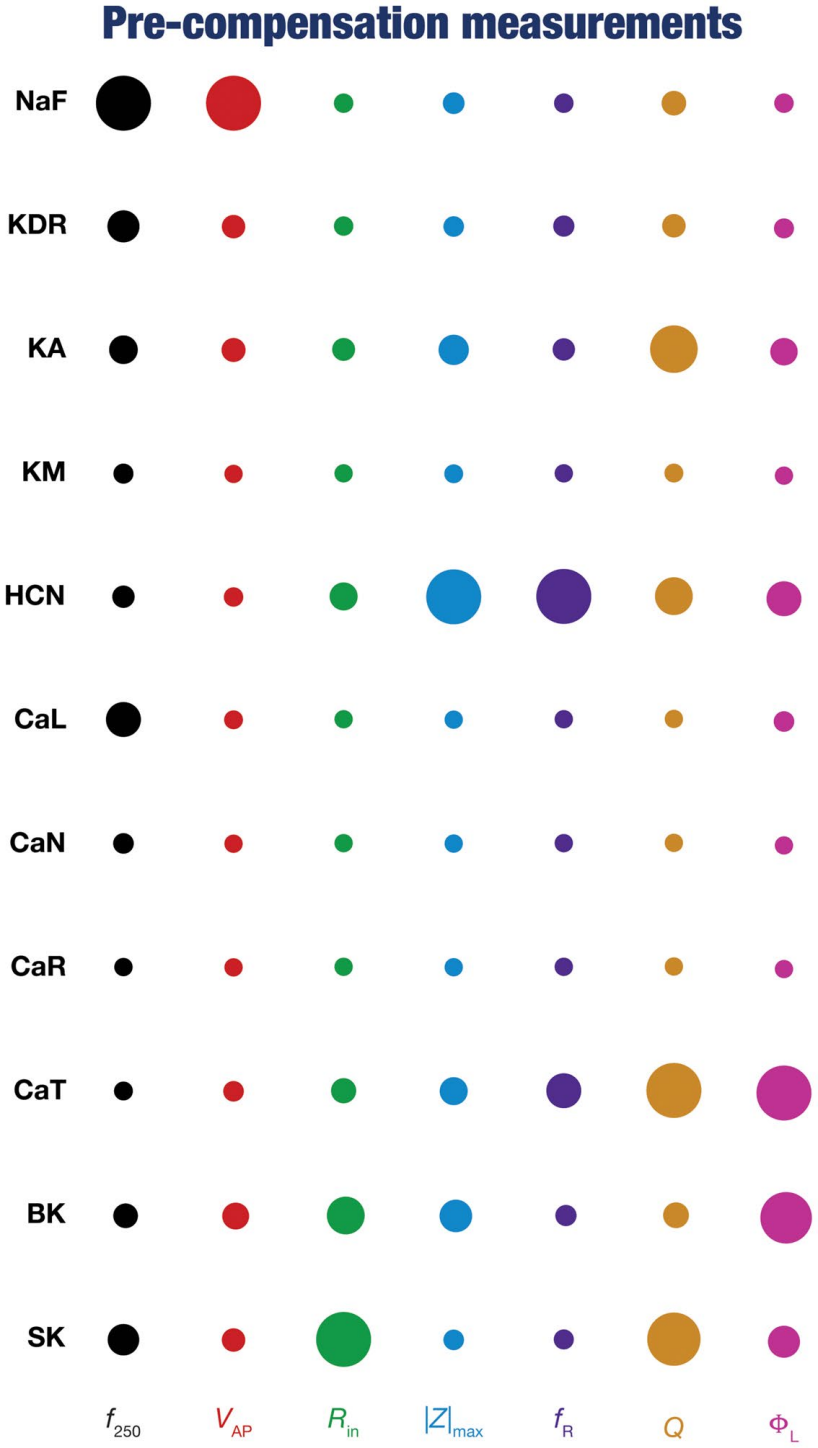
Diverse contributions of the different conductances to the seven physiological properties immediately after knockout. The relative impact of a particular conductance on different physiological properties is given by the diameter of the circle against that combination. The diameters of the circles have been normalized property-wise such that for each intrinsic property, the conductance corresponding to the largest/smallest diameters is responsible for the maximum/minimum impact, respectively, on that property. These representations were derived from the average percentage change in each physiological property consequent to deletion of each ion channel across all 78 models.

**Figure 5:**
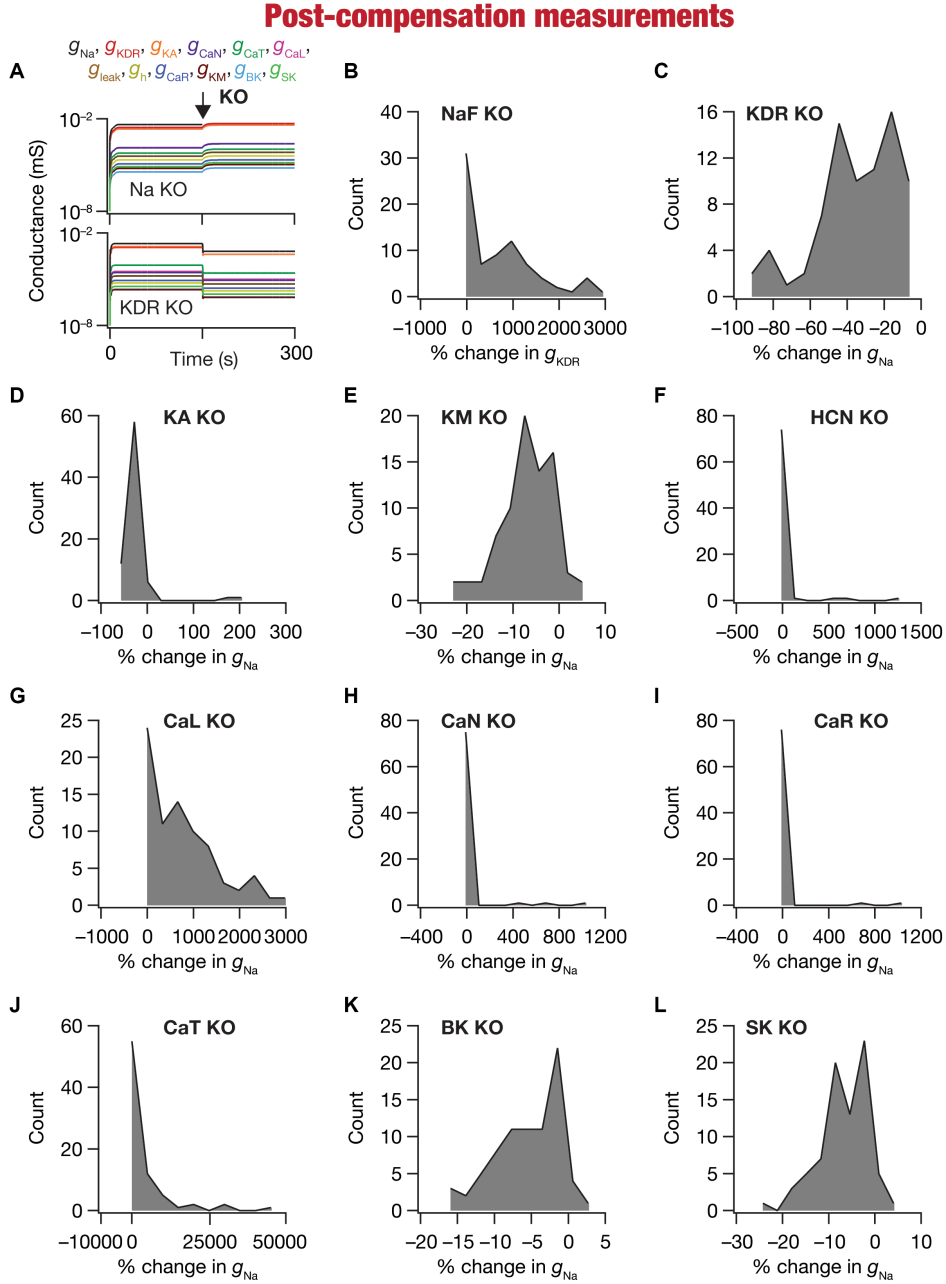
Heterogeneous post-knockout plasticity in individual conductances towards maintaining calcium homeostasis during theta-oscillatory activity across 78 models. **(A)** Example traces of the evolution of conductances for NaF (top) and KDR (bottom) knockouts. **(B)** Histogram of percentage changes in $g_{KDR}$ measured at steady state of compensation following virtual knockout of the Na conductance, computed with reference to the steady state value after evolution with θ-frequency oscillations. **(C–L)** Histogram of percentage changes in *g*_Na_ measured at steady state of compensation following virtual knockout of the 11 active ionic conductances [KDR **(C)**; KA **(D)**; KM **(E)**; HCN **(F)**; CaL **(G)**; CaN **(H)**; CaR **(I)**; CaT **(J)**; BK **(K)**; SK **(L)**], computed with reference to the steady state value after evolution with θ-frequency oscillations.

**Figure 6:**
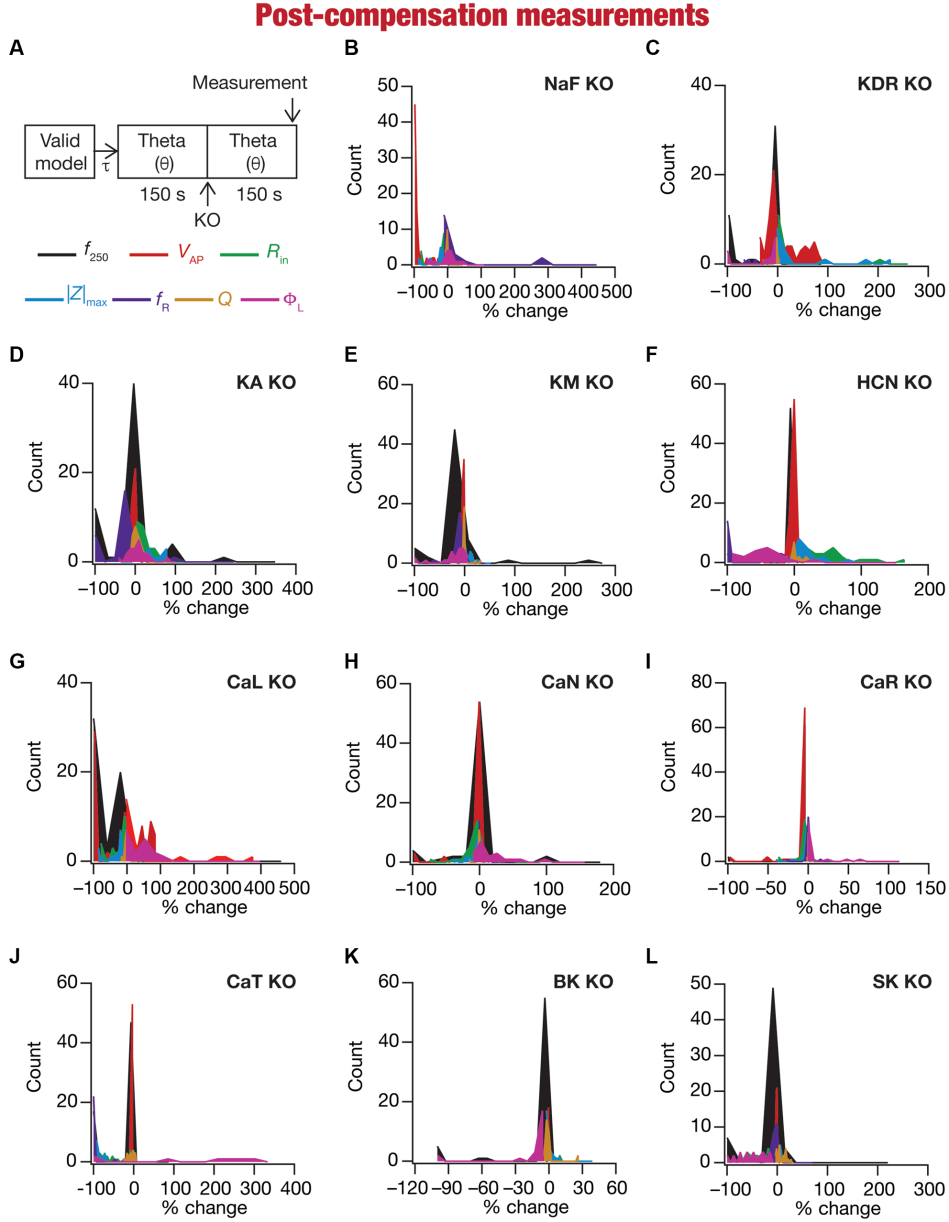
Heterogeneous post-knockout plasticity in the 7 physiological properties in maintaining calcium homeostasis during theta-oscillatory activity across 78 models. **(A)** Experimental design for assessing the effect of compensation after virtually knocking out ionic conductances on the seven intrinsic response properties (*f*_250_, *V*_AP_, *R*_in_, |Z|_max_, *f*_R_, *Q*, Φ_L_; color-coded) across 78 neuronal models. For each of the 78 valid neuronal models obtained from MPMOSS, ionic conductances were allowed to evolve towards achieving calcium homeostasis when afferent inputs were θ oscillations (θ). At steady state of this evolution (150 s), one of the conductances was “knocked-out” and the other conductances were allowed to continue evolving without any other perturbation until steady state was reached (150 s). The seven properties were measure at this steady state after compensation (post-compensation measurements). **(B–L)** Histogram of percentage changes in the 7 intrinsic response properties measured at steady state of compensation after knockout, computed with reference to the corresponding value of the intrinsic property measured at steady state of evolution after θ in response to compensation after knocking out the 11 active ionic conductances [Na **(B)**; KDR **(C)**; KA **(D)**; KM **(E)**; HCN **(F)**; CaL **(G)**; CaN **(H)**; CaR **(I)**; CaT **(J)**; BK **(K)**; SK **(L)**].

**Figure 7:**
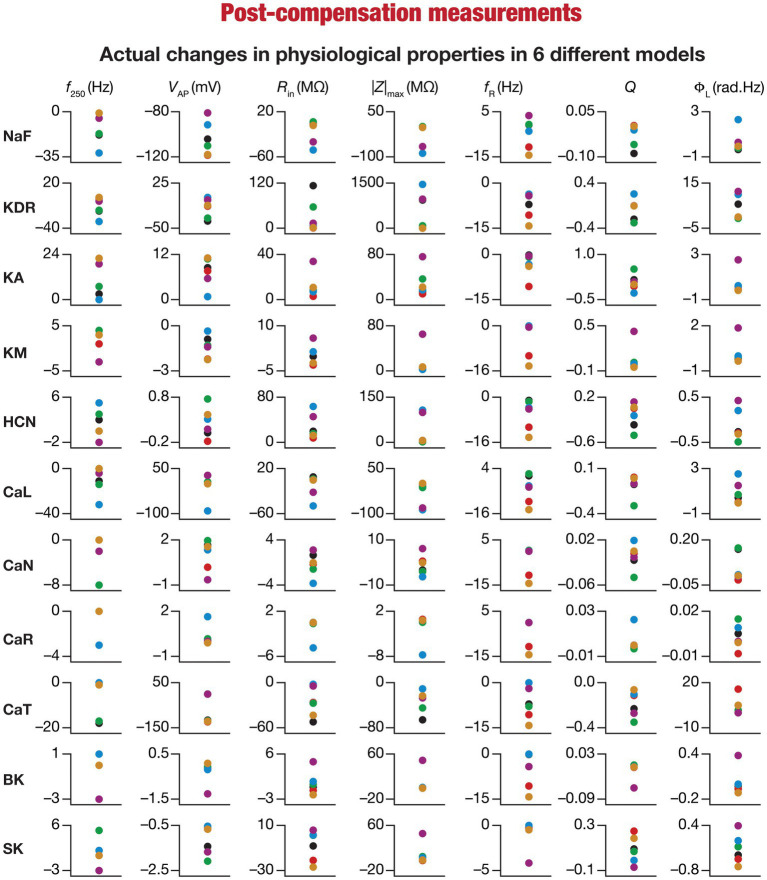
Heterogeneous post-knockout plasticity in the 7 physiological properties in maintaining calcium homeostasis during theta-oscillatory activity in 6 model neurons. Actual differences in seven intrinsic properties (*f*_250_, *V*_AP_, *R*_in_, |Z|_max_, *f*_R_, *Q*, Φ_L_) after compensation following virtual knockout of different conductances compared with steady state of theta-dependent evolution (θ) of 6 different valid models (color-coded).

**Figure 8:**
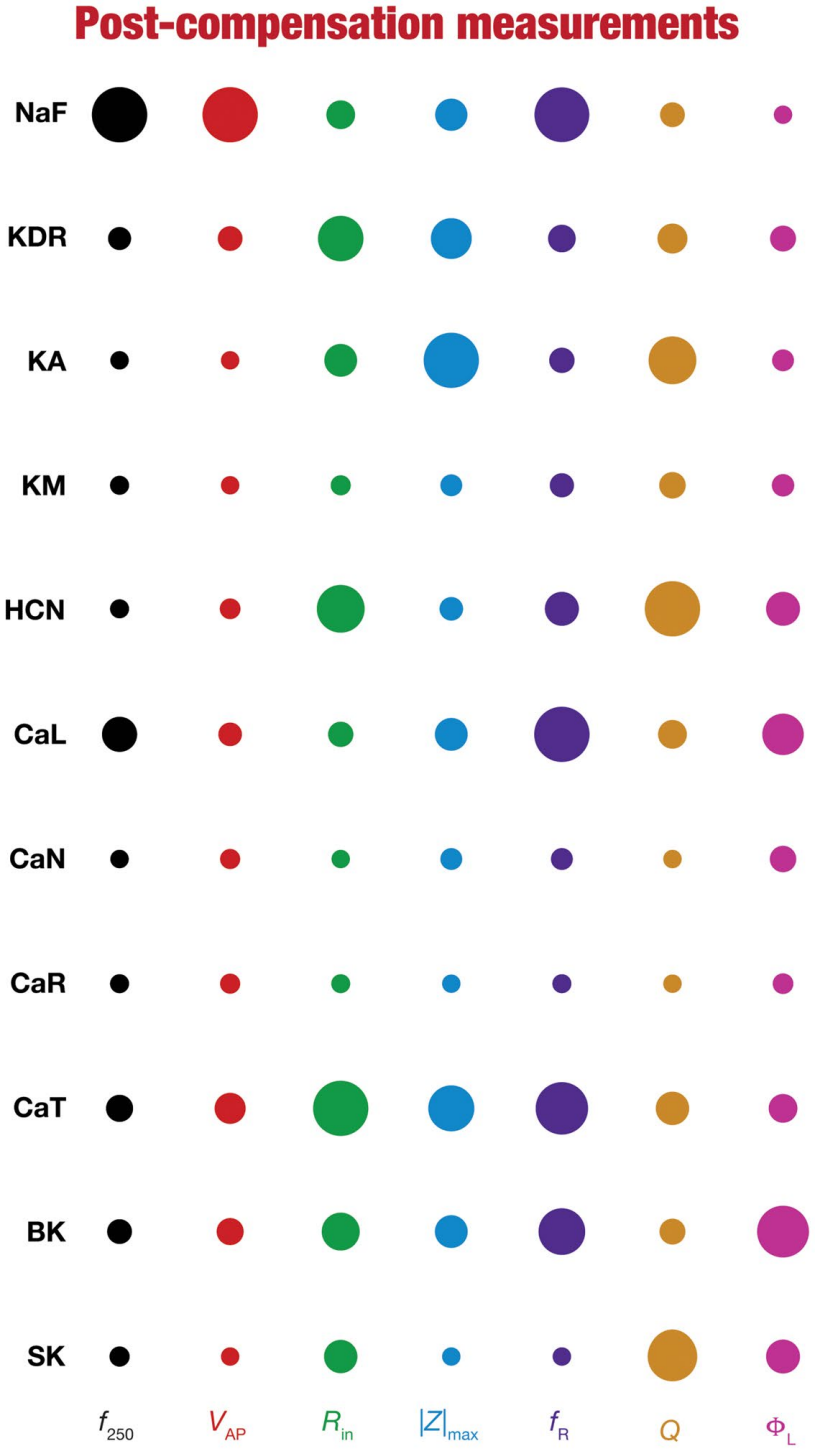
Post-knockout plasticity reveals diverse and differential impact of knockout of different conductances on the seven physiological properties. The impact of compensation following knockout of different conductances on the seven physiological properties is diverse. The relative impact of compensation consequent to knockout of a particular conductance on a particular intrinsic property is given by the diameter of the circle against that combination. The diameters of the circles have been normalized property-wise such that for each property, the conductance corresponding to the largest/smallest diameters is responsible for compensation that is pathological/restoring respectively, on that property. These representations were derived from the average percentage change in each physiological property following compensation after deletion of each ion channel across all 78 models.

**Figure 9:**
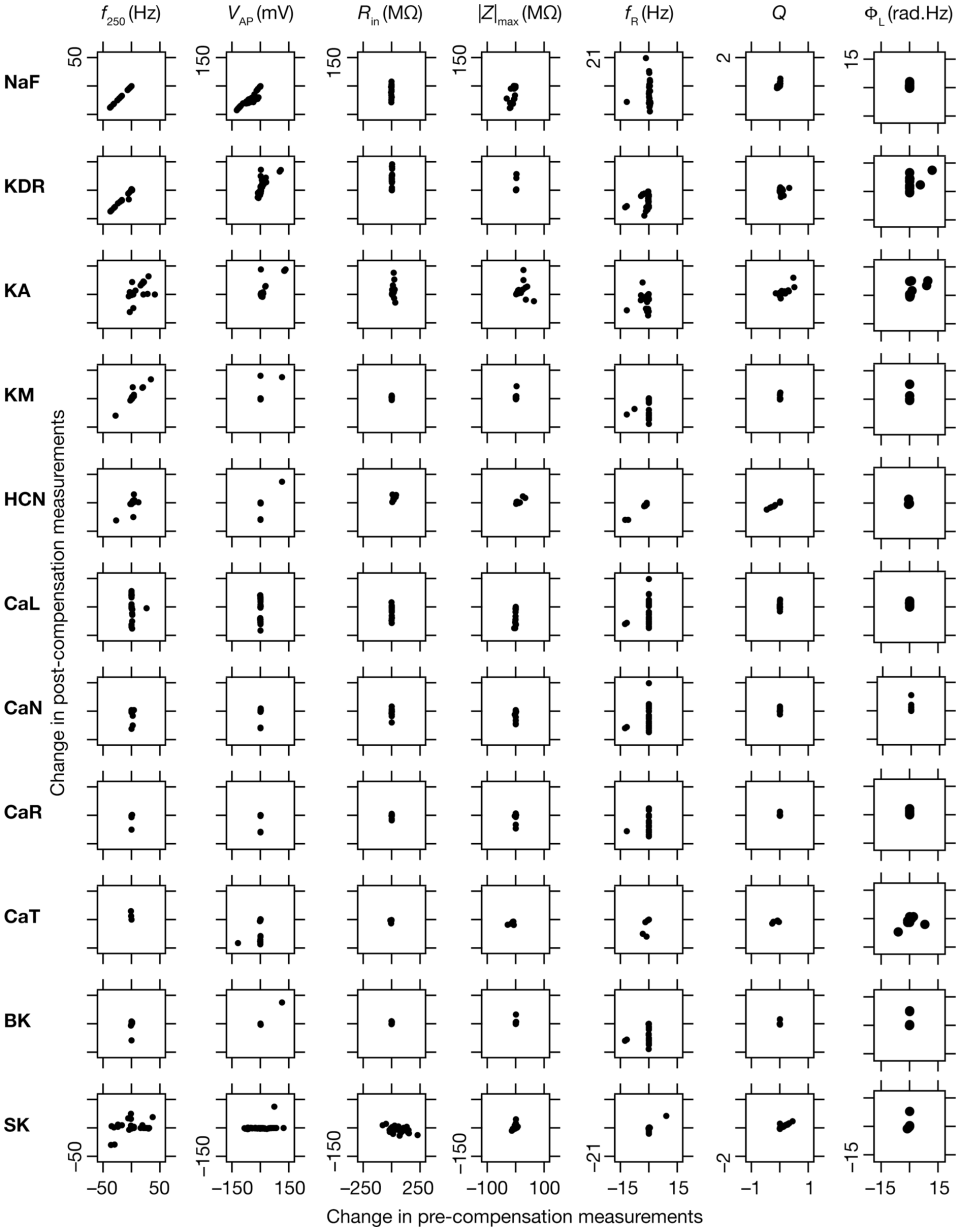
Relationship between properties changes before and after KO-driven plasticity. Each sub-figure is a scatter plot of the changes in a specific intrinsic property obtained post-compensation versus pre-compensation for the particular “knockout-intrinsic property” combination that it is plotted for. All plots for a particular physiological property have the same scale of axes.

Following our overall experimental plan for this study (Figure 1), we present our observations and analyses of changes at acute (i.e., immediately after knockout; Figures 2–4) and chronic (i.e., after steady-state of post-knockout plasticity; Figures 5–8) time points, finally comparing these changes (Figure 9) across this heterogeneous population of 78 model neurons.

### Heterogeneous impact of acute knockout of individual ion channels on neuronal intrinsic properties depended on the specific model, physiological property, and the deleted channel

We compared pre-compensation measurements with their pre-perturbation counterparts in simulations involving deletion of one of the 11 active ion channels across each of the 78 model neurons in the population (Figures 1, 2A). Neuronal intrinsic properties responded differentially to knockouts of specific ionic conductances, with the impact dependent on the specific physiological property and the conductance that was knocked out (Nolan et al., 2004; Chen et al., 2006; Narayanan and Johnston, 2008; Rathour and Narayanan, 2012a; O’Leary et al., 2014; Rathour and Narayanan, 2014; Anirudhan and Narayanan, 2015; Rathour et al., 2016; Mukunda and Narayanan, 2017; Basak and Narayanan, 2018b; Mittal and Narayanan, 2018; Rathour and Narayanan, 2019; Basak and Narayanan, 2020; Seenivasan and Narayanan, 2020; Mishra and Narayanan, 2021a,b; Roy and Narayanan, 2021; Mittal and Narayanan, 2022; Roy and Narayanan, 2022). Consistent with these observations, we found that channel knockouts had a heterogenous impact on different physiological properties across the 78 different models.

These heterogeneities were dependent on the model under consideration, the physiological property that was measured, as well as the specific channel that was deleted from that model. Specifically, deletion of the same ion channel resulted in very different changes in the same physiological property across different models (Figures 2B–L, 3). For instance, deletion of *T*-type calcium (CaT) channels reduced resonance frequency (*f*_R_) by different levels across different models (Figures 2J, 3). A similar trend was observed across different ion channels and physiological property combinations, whereby the deletion of a specific ion channel did not yield a unique change across all models but altered individual physiological properties by different values in different models (Figures 2B–L, 3). Second, the impact of channel deletion was differential across physiological properties, whereby some channels had a greater effect on certain physiological properties, with other channels exhibiting a weak impact on the same physiological properties. For instance, *f*_250_ and *V*_AP_ of the neuronal models were most affected by knocking out the NaF conductance (Figures 2B, 3), which is to be expected as NaF mediates a spiking current. However, *R*-type calcium (CaR) channels had very little impact on both these physiological properties (Figures 2I, 3). On the other hand, the HCN conductance had the maximum impact on |*Z*|_max_ and *f*_R_ of the neurons (Figure 2F), with the *N*-type calcium channel showing minimal impact (Figures 2H, 3). These observations also emphasize that changes in any given physiological property in a specific model were dependent on the particular channel that was being deleted from that model. For instance, across all models, ΦL showed minimal changes upon deletion of *R*-type calcium (CaR) channels (Figures 2I, 3), but was dominantly affected by HCN channel knockout (Figures 2F, 3). Assessing the 7 physiological properties across 6 randomly selected models and across the 11 different knockouts of active conductances, we noted that this variable impact of a particular knockout extended across all physiological properties in a model-dependent manner (Figures 2, 3).

To quantify the contributions of specific conductances to each of the seven intrinsic properties in our study, we adopted the analysis and visualization strategy employed earlier (Taylor et al., 2009; Rathour and Narayanan, 2014; Mukunda and Narayanan, 2017). We plotted circles against each conductance-physiological property combination such that the diameter of the circle represented the contribution of the conductance to that physiological property (Figure 4; *cf.* Figure 2). Specifically, a circle with larger diameter as an element of this matrix would imply a bigger impact of knocking out that conductance (row) on the specific intrinsic property (column). We found that these results, with acute knockouts, agreed with previously published experimental and computational results (Nolan et al., 2004; Chen et al., 2006; Narayanan and Johnston, 2008; Rathour and Narayanan, 2012a, 2014). For instance, spiking properties ( $f_{250}$ , $V_{AP}$ ) were heavily reliant on the sodium channel, impedance characteristics (|*Z*|_max_, $\Phi_{L}$ , *Q*, and *f*_R_) were dependent on resonating conductances (HCN and CaT). However, this did not imply a one-to-one relationship between the ion channels and physiological properties. There were other ion channels that did play a role in regulating individual physiological properties. As examples, CaL and SK channels played a critical role in setting the firing rate ( $f_{250}$ ), KA channels played modulatory role in setting resonance properties (Rathour and Narayanan, 2014, 2019; Rathour et al., 2016).

Together, these analyses provide consistent lines of evidence for many-to-many mappings between ion channels and physiological properties (Rathour and Narayanan, 2019; Goaillard and Marder, 2021; Mishra and Narayanan, 2021a,b; Yang and Prescott, 2023), emphasizing the expression of ion-channel degeneracy in these model neurons that evolved in an activity-dependent manner to achieve calcium homeostasis.

### Heterogeneous plasticity in ion channels mediated resilience of calcium homeostasis to knockout of individual channels

We observed the evolution of the 10 conductances (other than the one that was knocked out) in the 78 neuronal models and noted their values at steady state of calcium homeostasis after knockout of specific conductances (Figure 5A). Steady-state was allowed to evolve over a period of 150 s after knockout, with identical theta-frequency afferent activity impinging on the neuron as the 150 s period preceding knockout (Figures 1, 5A). Across all models and across all channels that were knocked out, we found that neurons were capable of achieving calcium homeostasis despite knockout of individual ion channels by altering other ion-channel conductances.

Changes in ion-channel conductances were computed from conductance values obtained after post-knockout steady-state (at 300 s; Figure 5A) with reference to respective values just before knockout (at 150 s; Figure 5A). As all changes are correlated within a given model (given that they are controlled by a single transcription factor), we plotted changes in a single conductance after post-knockout plasticity across all 78 models. We plotted changes in KDR conductance for the sodium knockout models (Figure 5B), and changes in sodium conductance for all the other channel knockouts (Figures 5C–L). Post-knockout plasticity in individual ion channels manifested pronounced heterogeneities, depending on the specific channel that was knocked out as well as on the specific model under consideration. For instance, very few models manifested plasticity in channel conductances after the knockout of either CaR or CaN channels (Figures 5H,I), but all models showed some plasticity with knockout of KDR channels (Figure 5C). In addition, in channels whose knockout resulted in considerable plasticity there was pronounced model-dependent heterogeneity even with the knockout of the same channels (Figures 5B,C).

Despite the pronounced heterogeneities, there was broad homogeneity in the direction of changes after knockout of specific conductances. Specifically, in a significant majority of models, knockout of channels that mediated inward (Figures 5B,F–J) and outward (Figures 5C–E,K,L) currents resulted in increases and decreases in other conductances, respectively, during the post-knockout emergence of calcium homeostasis. This is expected, because deletion of inward conductances typically reduce depolarization, thereby reducing cytosolic calcium concentration. Thus, the error signal in calcium with reference to the target calcium ( ${\left[Ca\right]}_{tgt}{\left[Ca\right]}_{i}$ ) that drives the mRNA changes (Eq. 21) is positive, together increasing all the conductance values (Eq. 22). For outward currents, on the other hand, the error signal is negative, thus reducing all the conductance values (All conductance values change in the same direction because a single transcription driven by the error signal and its sign govern plasticity of all conductances; Eq. 21–22). However, note that there were a small proportion of models that broke this general rule, emphasizing the role of global parametric structure in driving plasticity. For example, there were small proportions of models with knockout of KM (Figure 5E) or SK (Figure 5L) channels, which mediate an outward current, that showed an increase (not a decrease that would be typically expected from the deletion of an outward current) in the conductance value, implying that the error signal was positive in those models. This implies that the knockout of KM/SK channels in those models reduced cytosolic calcium (because of the loss of interactions with other channels that are present), together resulting in an overall increase of all conductance values. Additionally, knocking out voltage-gated calcium channels (especially CaL and CaT) resulted in significantly large increases in conductance values, given that these channels directly contributed to the maintenance of calcium homeostasis before they were knocked out (Figures 5G–J).

Together, our analyses demonstrated that calcium homeostasis was resilient to deletion of individual ion channels. Although calcium homeostasis was perturbed in most models after the knockout of individual channels, calcium homeostasis was reestablished through model- and knockout-dependent plasticity in the other ion channels. In most knockout models, our analyses pointed to a directional preference in the conductance change based on whether the deleted channel mediated inward or outward currents. However, there were small proportions of models where this preference was reversed, pointing to a role for global structure in regulating calcium dynamics.

### Heterogeneous impact of post-knockout calcium homeostasis on neuronal intrinsic properties

Ideally, compensation should result in all intrinsic properties of the neuron being preserved/restored. However, there are lines of evidence to suggest that compensation may either preserve/restore a function or be pathological (O’Leary et al., 2014; Yang et al., 2022). Consistent with this, we observed pronounced heterogeneity in the impact of post-knockout plasticity towards achieving calcium homeostasis, in a manner that was dependent on the specific model, the conductance that was knocked out, and the model under consideration (Figures 6B–L, 7). For each knockout, there were examples of post-knockout compensation leading to preservation of intrinsic properties and of post-knockout plasticity leading to large changes in intrinsic properties.

To visualize the impact of compensation on the intrinsic properties for each of the 11 virtual knockouts of active conductances, we constructed the circle-based representation we had employed earlier in Figure 4. We found pronounced heterogeneity in the impact of post-knockout plasticity of different channels on the 7 physiological properties (Figure 8). Importantly, there was no direct relationship of these changes to whether a specific ion channel was implicated in mediating a given physiological property (Figure 8). For instance, although the NaF and CaL channels have not been directly implicated in mediating subthreshold resonance (also evidenced from Figure 4), we noted that compensatory changes in other channels after either of these two ion channels were knocked out resulted in pronounced changes to *f*_R_ (Figure 8). Importantly, post-compensatory measurements associated with several models with different knockouts were not within the experimental ranges typically measured from CA1 pyramidal neurons (Table 2), reemphasizing the dissociation between calcium homeostasis and functional homeostasis. Specifically, although all models across all knockouts achieved calcium homeostasis efficiently, neither the conductance values of different ion channels (Figure 5) nor the functional properties (Figures 6–8) were maintained at specific values (Tables 1, 2) after the post-knockout evolution of conductances.

Together, these results demonstrate that ion-channel conductances and neuronal intrinsic properties could undergo significant plasticity after knockout of channel conductances towards maintaining homeostasis, with the strength of changes varying significantly across models and physiological properties for each of the 11 knockouts (of active conductances). There was considerable heterogeneity in post-knockout plasticity of different ion channels towards achieving calcium homeostasis, introducing changes in physiological properties that were not directly connected to the channel that was being knocked out. Thus, post-knockout plasticity geared towards maintaining homeostasis introduced off-target effects on other channels that were not perturbed (as part of deleting single channels). Post-knockout plasticity spanning several channels also manifested considerable neuron-to-neuron variability in how different channels and physiological properties responded to knockout of individual ion channels.

### Diversity in the relationship between acute and chronic changes in intrinsic properties consequent to individual channel knockouts

When assessed immediately after knockout of specific ionic conductances, neuronal intrinsic properties responded differentially to the knockout, with the impact dependent on the specific physiological property and the conductance that was knocked out (Figures 2–4). Additionally, post-knockout compensation towards achieving calcium homeostasis could either restore function or introduce large changes compared to the pre-perturbation measurements. Together, with reference to a single physiological property, we could potentially have 4 classes of neurons, with 2 types of changes (change vs. no change) each at acute knockout and at steady state after post-knockout evolution (O’Leary et al., 2014). To study these categories of neurons, we plotted the changes in the physiological property obtained after compensation against their respective pre-compensation changes for each of the 11 knockouts and 7 intrinsic properties (Figure 9). We noted that even for the same channel knockout, neurons that belonged to one of the four classes with reference to one physiological property did not fall into the same class with reference to another physiological property. For instance, although *R*_in_ was very robust to acute knockout of NaF channels, post-knockout compensations after NaF knockout resulted in significant changes to *R*_in_; however, Φ_L_ was very robust to acute knockout of NaF and did not undergo significant changes during post-knockout plasticity as well. This was observed across knockouts, where different physiological properties fell into different classes of acute vs. chronic changes. Notably, although several physiological properties did not change considerably with acute knockout of voltage-gated calcium channels, post-knockout plasticity in intrinsic properties (towards achieving calcium homeostasis) was relatively higher after knockout of calcium channels (Figure 9).

## Discussion

The key conclusion of this study is that there are pronounced heterogeneities in the acute as well as chronic impact of knocking out individual channels on intrinsic properties of neurons undergoing homeostatic intrinsic plasticity. The specific impact depended critically on the conductance being knocked out, the intrinsic property under consideration, and the neuronal model in question. The use of a heterogeneous population of 78 neurons, as opposed to a single hand-tuned model, efficaciously brought forth model-dependent heterogeneities in the acute as well as chronic impact on intrinsic properties. Our analyses provide further lines of evidence for many-to-many mappings between ion channels and physiological properties, emphasizing the expression of ion-channel degeneracy in these model neurons across different physiological properties and their combinations. Importantly, our analyses demonstrate that calcium homeostasis is resilient to deletion of individual ion channels. Although calcium homeostasis was perturbed in most models after the knockout of individual channels, target calcium levels were achieved through heterogeneous homeostatic plasticity involving other ion channels. Through such homeostatic intrinsic plasticity, ion-channel conductances and neuronal intrinsic properties underwent significant plasticity after knockout of individual channels, translating to off-target effects that are unrelated to the deleted ion channels.

### Functional dissociation in different forms of homeostasis

Our assessments following individual deletion of each of the 11 active ion channels and measuring seven distinct intrinsic properties elucidated the channel- and function-dependence of the assessed impact of channel knockouts. In demonstrating these dependencies and the consequent heterogeneities, our analyses emphasized the clear dissociation between different forms of homeostasis (Srikanth and Narayanan, 2015). Specifically, our analysis clearly demonstrates that calcium homeostasis can be resilient to knockout of individual channels and may be achieved through heterogeneous plasticity in the other ion channels. However, achieving calcium homeostasis did not necessarily translate to restoration of any of the several functional measurements to their original values. Thus, function-specific analyses spanning several different physiological measurements becomes essential in assessing the impact of knockouts. There have been several studies spanning the cellular and networks scales of neuroscience that have incorporated the goal of achieving *multiple objectives* into model search processes (Bhalla and Bower, 1993; Foster et al., 1993; Goldman et al., 2001; Golowasch et al., 2002; Prinz et al., 2003, 2004; Keren et al., 2005; Achard and De Schutter, 2006; Tobin et al., 2006; Druckmann et al., 2007; Reid et al., 2007; Hobbs and Hooper, 2008; Weaver and Wearne, 2008; Menon et al., 2009; Taylor et al., 2009; Bahl et al., 2012; Zhao and Golowasch, 2012; Rathour and Narayanan, 2012a,b, 2014, 2019; Friedrich et al., 2014; Anirudhan and Narayanan, 2015; Srikanth and Narayanan, 2015; Van Geit et al., 2016; Beining et al., 2017; Mukunda and Narayanan, 2017; Neymotin et al., 2017; Gouwens et al., 2018; Migliore et al., 2018; Mittal and Narayanan, 2018, 2022; Basak and Narayanan, 2018b, 2020; Mishra and Narayanan, 2019; Jain and Narayanan, 2020; Navas-Olive et al., 2020; Seenivasan and Narayanan, 2020; Pallasdies et al., 2021; Roy and Narayanan, 2021, 2022; Deistler et al., 2022; Jedlicka et al., 2022; Shridhar et al., 2022; Sinha and Narayanan, 2022; Yang et al., 2022; Nagaraj and Narayanan, 2023). This framework of achieving multiple physiological goals should be invoked in characterizing and assessing the impact of any perturbation as well. Focus on a single functional outcome and a rescue that is specific to that single physiological property would ignore the multi-faceted impact of channel perturbations, involving distinct acute and chronic components. In such assessments involving multiple functional measurements, it is critical to account for neuron-to-neuron heterogeneities in the expression profile of constitutive components, the ubiquitous manifestation of degeneracy in achieving multiple physiological goals, and ion-channel pleiotropy that enables the same channel to execute different functions in different contexts.

In such analyses involving achievement of multiple objectives, care should be taken to (i) identify sloppy parameters/dimensions in the parametric space; and (ii) assess whether the multiple objectives are indeed distinct from each other. First, with reference to the parametric space, if the functional outcome of a system is insensitive to changes in a parameter or in direction of changes in specific parametric combinations, the overall system is considered sloppy (Daniels et al., 2008; O’Leary et al., 2015). It is important to note that sloppiness is defined based on functional outcomes. For example, although neuronal morphology might be a sloppy parameter with reference to electrical functions of neurons (Otopalik et al., 2017a,b, 2019), it plays critical roles with reference to compartmentalization of second messengers and other signaling molecules (Koch and Zador, 1993; Zador and Koch, 1994; Berridge, 2006; Neves et al., 2008; Newpher and Ehlers, 2008; Rangamani et al., 2013; Basak and Narayanan, 2018a, 2020). In a multi-objective framework, it is essential to identify the sloppy dimensions/parameters in the parametric space with reference to the specific objectives that are being achieved. In addition, such analyses should also explore parametric cross-dependencies. For instance, there could be two parameters that identically alter all the measured objectives. In such a scenario, there could be a strong dependence between the two parameters, making the independent parametric space smaller than the actual parametric space.

Second, with reference to measurement space, it is critical to identify whether the chosen objectives (with the multi-objective framework) indeed require tradeoffs involving the set of parameters that are being used for optimization. As an example, consider a neuron where there are no active subthreshold conductances rendering the subthreshold properties to be dependent solely on passive neuronal properties. In such a neuron, the input resistance of the neuron and the maximal impedance amplitude would essentially be very strongly correlated to each other. Thus, a multi-objective procedure involving input resistance and maximal impedance amplitude in such a neuron would essentially map on to a single functional measurement. Thus, the cross-dependencies of the different objectives should be carefully analyzed to assess whether there are indeed tradeoffs required in achieving these objectives. In the absence of such analyses, it is possible that the multiple objectives just map on to a single objective without tradeoffs or involve objectives that are insensitive to parametric choices (or their chosen ranges).

### Heterogenous secondary and off-target effects of homeostatic intrinsic plasticity following ion-channel deletion

Our analyses show that a specific physiological characteristic could be resilient to acute blockade of a specific channel, but under chronic knockout scenarios where calcium homeostasis is actively achieved, the same physiological property could undergo considerable plasticity. Such acute-chronic dissociation arises because of homeostatic intrinsic plasticity spanning other ion channels, thus implying that chronic changes would not be limited to the physiological properties that were originally mediated by the channel that was knocked out. These results have significant ramifications for the interpretation of results obtained with animals subjected to genetic knockouts. Specifically, these results imply that a thorough analysis of several neuronal and network physiological properties need to be performed on knockout animals, with special emphasis on compensatory changes in all physiological properties (across the neuron), before assigning a causal role for an ion channel or a receptor in a specific behavioral task or a physiological phenomenon.

In assessing neural circuits, individual components have been assigned to have instructive vs. permissive roles with clear distinctions made about acute vs. chronic impacts of deleting individual components (Wolff and Olveczky, 2018). Our analyses clearly show that such analyses are not limited to neural circuit components, but also components of individual neurons. The analysis of the impact of ion channels on specific physiological property should clearly account for post-deletion plasticity as well as the many-to-many relationship between channels and cellular physiology. In addition, just as in neural circuits (Wolff and Olveczky, 2018), the strong voltage- and/or calcium-linked feedback loops mediated by individual ion channels point to a complex role for each ion channel in regulating neuronal physiology. Thus, the emphasis should be placed on global structure of the parametric space rather than on individual ion channels, whereby degeneracy (many-to-one) and pleiotropy (one-to-many) are explicitly factored in the mapping between ion channels and functional outcomes (Goldman et al., 2001; Marder and Bucher, 2007; Marder, 2011, 2012; Marder and Taylor, 2011; Zhao and Golowasch, 2012; Rathour and Narayanan, 2012a,b, 2014, 2019; Marder et al., 2014; O’Leary, 2018; Goaillard and Marder, 2021; Mishra and Narayanan, 2021c; Yang and Prescott, 2023).

Across cell types, the impact of acute blockade of ion channels using pharmacological agents on neuronal physiology is measurement-dependent and manifests pronounced heterogeneities even for a given physiological property (Grashow et al., 2010; O’Leary et al., 2014; Rathour and Narayanan, 2014; Mukunda and Narayanan, 2017; Mittal and Narayanan, 2018, 2022; Basak and Narayanan, 2018b, 2020; Jain and Narayanan, 2020; Seenivasan and Narayanan, 2020; Mishra and Narayanan, 2021a,b). Such heterogeneous acute impact could trigger heterogeneous homeostatic plasticity towards reversing such perturbations, leading to heterogeneities in homeostasis-driven off-target and secondary impact of such perturbations (Ransdell et al., 2012). Thus, interpretation of causal manipulation of outcomes needs to account for acute heterogeneities as well as heterogeneities in homeostatic compensations. As the cumulative observed outcome is a combination of acute impacts and post-knockout plasticity, to attribute the final outcome to a single ion channel would be inappropriate (Chan et al., 2007; Andrasfalvy et al., 2008; Chen et al., 2010; Ransdell et al., 2012; O’Leary et al., 2014; Yang et al., 2022). In experiments involving pharmacological agents for blockade of ion channels (or in inducing other forms of perturbation), it is essential to account for non-specificities associated with these pharmacological agents that alter components other than the targeted molecule.

Our results imply that a thorough analysis of several neuronal and network physiological properties need to be performed on genetic knockout animals, especially when assigning a causal role for an ion channel or a receptor. This is important because the observed change in the behavioral task or in a physiological phenomenon may just be a reflection of the compensatory changes in *other* channels/receptors of knockout animals, with the channel/receptor that was knocked out playing little or no role in wild-type animals (Chan et al., 2007; Andrasfalvy et al., 2008; Chen et al., 2010). Together, these observations call for a detailed assessment of compensatory mechanisms in each neuronal subtype (across the somato-dendritic-axonal arbor) and their networks in knockout animals, accounting for heterogeneities as well as degeneracy. Such analyses involving spatiotemporal interactions among channels across the neuronal morphology is essential before a causal link is established between a channel/receptor and a behavioral task or a physiological phenomenon.

### Limitations and future directions

There are several simplifying assumptions that are part of the analysis presented here and should be rectified in future assessment of the question of the impact of perturbations on physiological properties and homeostasis. First, we employed a single-compartmental model in our analyses which did not account for the morphological complexity of the dendritic arbor or the functional maps that express in these dendrites (Johnston and Narayanan, 2008; Nusser, 2009; Narayanan and Johnston, 2012; Hanus and Schuman, 2013; Rathour and Narayanan, 2014). It is essential that morphologically realistic neuronal models are built with active dendritic components, so that plasticity and homeostasis are dependent on complex interactions between afferent activity patterns, back-propagating action potentials, dendritic spikes, and plateau potentials. Deletion of individual channels associated with such a morphologically realistic model population, in the presence of homeostatic plasticity mechanisms, would provide avenues for exploring differential somatodendritic plasticity profiles in effectuating homeostasis after channel deletion. Accounting for neuronal morphology and size, including the differential expression channel proteins and channel-modulating enzymes would also allow for the expansion of the framework of homeostatic plasticity to local vs. global activity levels (Turrigiano and Nelson, 2004; Rabinowitch and Segev, 2006a,b, 2008; Nelson and Turrigiano, 2008; Turrigiano, 2011; Gorur-Shandilya et al., 2020). Such analyses should be performed in a framework where morphologically realistic neurons and their networks are subjected to different forms of behaviorally relevant afferent activity (not just theta oscillations), with homeostatic plasticity spanning channels and receptors across the somatodendritic arbor. It is also important that plasticity is not confined to mere maintenance of homeostasis, but is geared towards energy efficiency, efficient information coding, and continual learning in response to perpetual changes in environmental activity patterns (Narayanan and Johnston, 2012; Srikanth and Narayanan, 2015; Rathour and Narayanan, 2019; Mishra and Narayanan, 2021c; Deistler et al., 2022; Jedlicka et al., 2022; Seenivasan and Narayanan, 2022; Yang et al., 2022).

Second, our analyses assume that all channel conductances are regulated by a single transcription factor (O’Leary et al., 2014; Srikanth and Narayanan, 2015), an assumption that significantly oversimplifies the complexities of neuronal transcription, where multiple transcription factors coexist (Bading et al., 1993; Dolmetsch, 2003; Lein et al., 2007; Alberini, 2009). This assumption implies that proportions of changes in channel conductances are correlated, also accounting for our observation that all conductances changed in a specific direction with knockout of inward/outward currents (Figure 5). Future theoretical studies should account for multiple calcium-dependent enzymes and multiple transcription factors coupled through established signaling motifs, including negative feedback mechanisms (Thattai and van Oudenaarden, 2001; Losick and Desplan, 2008; Yu et al., 2008; Kotaleski and Blackwell, 2010; Cheong et al., 2011). Such analyses should account for relationships between the different transcription factors, mRNAs, and channel conductances across the somatodendritic arbor of single hippocampal neurons, the signaling cascades that regulate these channel properties, and heterogeneities in each component. Homeostatic plasticity obtained in such models where all channels and all forms of plasticity are accounted for could then be assessed for the manifestation of plasticity manifolds (beyond the simple correlation-based manifolds that emerge with single transcription factors) that impose structured rules on plasticity mediating homeostasis (Mishra and Narayanan, 2021c). In this context, while baseline calcium homeostasis is important, it is also essential to account for how neurons respond to plasticity-inducing stimuli. Specifically, the maintenance of calcium homeostasis does not necessarily translate to similar calcium profiles in response to plasticity-inducing stimuli. Thus, if plasticity homeostasis were also to be accounted for, then specific plasticity inducing patterns, resultant calcium responses, and plasticity patterns should also be accounted for in analyzing model outcomes (Ashhad and Narayanan, 2013; Honnuraiah and Narayanan, 2013; Anirudhan and Narayanan, 2015; Mukunda and Narayanan, 2017; Rathour and Narayanan, 2019; Shridhar et al., 2022).

Finally, our analyses assume deterministic channel models and deterministic signaling evolution, whereas stochasticity is ubiquitous across all biological systems. The impact of extrinsic and intrinsic noise on transcription, translation, signaling cascades, synaptic transmission, and channel physiology need to be carefully accounted for in assessing the impact of channel deletion on neural encoding and homeostasis within a degeneracy framework (Edelman and Gally, 2001; Rao et al., 2002; Swain et al., 2002; Kitano, 2007; Bhalla, 2014; Selimkhanov et al., 2014; Rathour and Narayanan, 2019; Mishra and Narayanan, 2021c; Mittal and Narayanan, 2022; Seenivasan and Narayanan, 2022). In this context, a recent study demonstrates that the simple model governed by a single calcium-dependent transcription factor results in unbounded biophysical parameters in the presence of colored noise (Franci et al., 2020). A revised model introduces additional post-transcriptional molecular regulatory networks to stabilize the network, while also reconciling between variability and robustness (Franci et al., 2020). Along similar lines, (Yang et al., 2022) demonstrated that the presence of noise affected ion-channel correlations in a simple model involving regulation of firing rate, but correlations were relatively unaffected by noise when firing rate and energy efficiency were co-regulated. Together, the ubiquity of biological noise warrants careful stability analysis of system evolution and there are lines of evidence that additional factors are required for stabilization in the presence of noise (Franci et al., 2020; Yang et al., 2022). Future studies should assess the impact of channel knockouts in networks endowed with the rich biological regulatory mechanisms that robustly stabilize homeostasis in the presence of noise and variability. In addition to stochasticity in these different components, afferent inputs to the neurons should also account for variability (both in amplitude and frequency) and state-dependence in theta-frequency oscillatory inputs (Buzsaki, 2002; Buzsaki and Moser, 2013; Colgin, 2013).

## Data availability statement

The original contributions presented in the study are included in the article/Supplementary material, further inquiries can be directed to the corresponding author.

## Author contributions

SS and RN designed research and wrote the paper. SS performed research and analyzed data. All authors contributed to the article and approved the submitted version.
