# Supplementary material for: Heterogeneous off-target impact of ion-channel deletion on intrinsic properties of hippocampal model neurons that self-regulate calcium
Source: Front Cell Neurosci. Author manuscript; Available in PMC 2023 Nov 4. (PMC10613471; doi:10.3389/fncel.2023.1241450)
Supplement: Supplementary Material [file EMS190319-supplement-Supplementary_Material.zip › Data Sheet 1.docx]

Supplementary Material

Heterogeneous off-target impact of ion-channel deletion on intrinsic properties of hippocampal model neurons that self-regulate calcium

Sunandha Srikanth and Rishikesh Narayanan^*^

*** Correspondence:** Rishikesh Narayanan, rishi@iisc.ac.in

# Supplementary Data

A ZIP file containing the codes used for the simulations reported in this study, along with parametric combinations used, are being uploaded separately as a supplementary file associated with this manuscript.
